# Reciprocal effects of alpha-synuclein aggregation and lysosomal homeostasis in synucleinopathy models

**DOI:** 10.1186/s40035-023-00363-z

**Published:** 2023-06-13

**Authors:** Alice Drobny, Fanni Annamária Boros, Denise Balta, Susy Prieto Huarcaya, Deniz Caylioglu, Niyeti Qazi, Julia Vandrey, Yanni Schneider, Jan Philipp Dobert, Caleb Pitcairn, Joseph Robert Mazzulli, Friederike Zunke

**Affiliations:** 1 Department of Molecular Neurology, University Hospital Erlangen, Friedrich-Alexander University Erlangen-Nürnberg, 91054 Erlangen, Germany; 2 Institute of Biochemistry, Christian-Albrechts-University Kiel, Kiel, Germany; 3 The Ken and Ruth Davee Department of Neurology, Northwestern University Feinberg School of Medicine, Chicago, IL 60611 USA

**Keywords:** Dopaminergic neurons, iPSC-derived models, Lysosome, Parkinson’s disease, Protein trafficking, Synucleinopathy

## Abstract

**Background:**

Lysosomal dysfunction has been implicated in a number of neurodegenerative diseases such as Parkinson’s disease (PD). Various molecular, clinical and genetic studies have highlighted a central role of lysosomal pathways and proteins in the pathogenesis of PD. Within PD pathology the synaptic protein alpha-synuclein (αSyn) converts from a soluble monomer to oligomeric structures and insoluble amyloid fibrils. The aim of this study was to unravel the effect of αSyn aggregates on lysosomal turnover, particularly focusing on lysosomal homeostasis and cathepsins. Since these enzymes have been shown to be directly involved in the lysosomal degradation of αSyn, impairment of their enzymatic capacity has extensive consequences.

**Methods:**

We used patient-derived induced pluripotent stem cells and a transgenic mouse model of PD to examine the effect of intracellular αSyn conformers on cell homeostasis and lysosomal function in dopaminergic (DA) neurons by biochemical analyses.

**Results:**

We found impaired lysosomal trafficking of cathepsins in patient-derived DA neurons and mouse models with αSyn aggregation, resulting in reduced proteolytic activity of cathepsins in the lysosome. Using a farnesyltransferase inhibitor, which boosts hydrolase transport via activation of the SNARE protein ykt6, we enhanced the maturation and proteolytic activity of cathepsins and thereby decreased αSyn protein levels.

**Conclusions:**

Our findings demonstrate a strong interplay between αSyn aggregation pathways and function of lysosomal cathepsins. It appears that αSyn directly interferes with the enzymatic function of cathepsins, which might lead to a vicious cycle of impaired αSyn degradation.

**Graphical abstract:**

Lysosomal trafficking of cathepsin D (CTSD), CTSL and CTSB is disrupted when alpha-synuclein (αSyn) is aggregated. This results in a decreased proteolytic activity of cathepsins, which directly mediate αSyn clearance. Boosting the transport of the cathepsins to the lysosome increases their activity and thus contributes to efficient αSyn degradation.
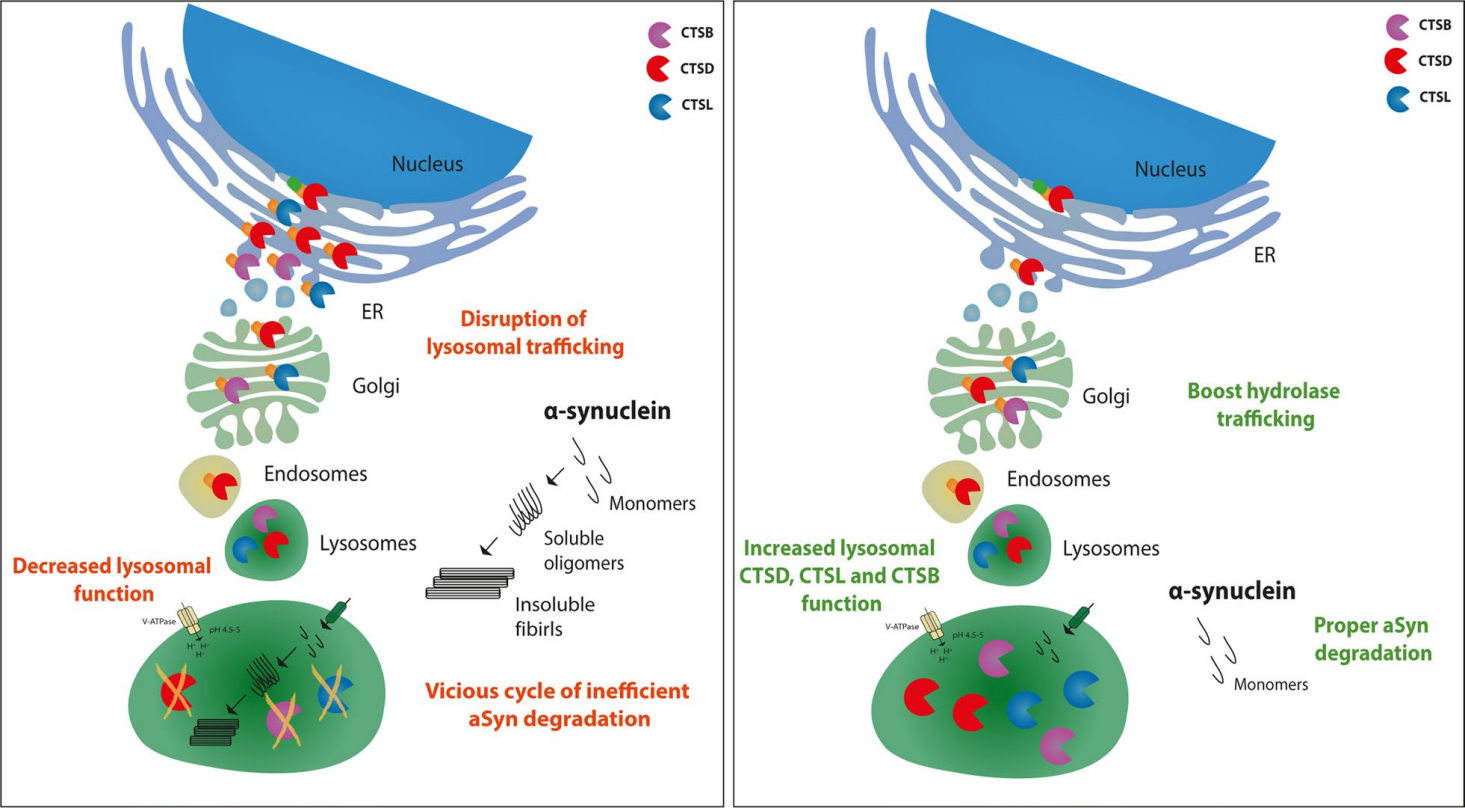

**Supplementary Information:**

The online version contains supplementary material available at 10.1186/s40035-023-00363-z.

## Background

Numerous neurodegenerative diseases are characterized by the progressive accumulation and misfolding of specific proteins into insoluble inclusions or aggregates. In Parkinson’s disease (PD) and other synucleinopathies, the soluble pre-synaptic protein alpha-synuclein (αSyn) is found to be intracellularly accumulated within inclusions, termed Lewy bodies and neurites [1]. These pathological αSyn forms persist in PD patients over the course of the disease and result in the demise of dopaminergic (DA) neurons in the substantia nigra (SN) [2]. Rare familial genomic predispositions can lead to elevated αSyn levels, accelerating aggregation and onset of disease. These include missense mutations in the *SNCA* gene like p.A53T [3] and duplications or triplications of the gene [4–6] resulting in accelerated αSyn aggregation and early onset of disease. In addition to abnormal synthesis, the insufficient clearance of αSyn by degradative systems within the cell has been implicated in PD [7]. Protein homeostasis, also referred to as proteostasis, is a complex network responsible for a delicate balance of protein synthesis and turnover to ensure the stability and functional features of any cell [8]. This process is critical in neurons as they are post-mitotic cells and thus rely heavily on available degradative organelles to clear substrates. The lack of substrate dilution by cell division renders neurons vulnerable to cellular toxicity and degeneration [7, 9].

A crucial mechanism involved in the recycling and degradation of accumulated material is the autophagy-lysosome pathway (ALP) [10], which has been shown to be involved in the clearance of pathological αSyn [11, 12]. Hence, it is not surprising that lysosomal dysfunction has been implicated not only in PD but also in numerous neurodegenerative disorders [13, 14]. In turn, the majority of lysosomal storage disorders (LSD), which are caused by the loss-of-function of distinct lysosomal enzymes, incorporate neurodegenerative symptoms, indicating the importance of a proper lysosomal function for cell homeostasis [15]. Genome-wide association studies in PD patients have determined several risk loci within genes known to cause LSDs [16]. For instance, mutations of the *GBA1* gene encoding the lysosomal hydrolase beta-glucocerebrosidase (GCase) not only cause the LSD Gaucher disease but are also one of the highest genetic risk factors for developing PD [17, 18]. The loss-of-function of the lysosomal protease cathepsin D (CTSD) has even more pronounced consequences, since it leads to neuronal ceroid lipofuscinoses type 10 (NCL-10), a severe LSD associated with congenital mental retardation, or juvenile neurodegeneration in humans and other mammalian species [19]. In vivo studies also suggest that not only the knockout (KO) of CTSD but also double KO of the lysosomal proteases cathepsin B (CTSB) and cathepsin L (CTSL) can cause a similar NCL-10 phenotype [20]. Strikingly, mutations in CTSD [16] and CTSB [21] have been identified as genetic risk loci for developing PD.

The aspartic CTSD and the cysteine CTSB and CTSL are the most abundant among lysosomal proteases and are particularly found in the brain [22]. They are known to degrade several important neuronal substrates, and thus are implicated in numerous neurodegenerative disorders [23]. Most importantly, a clear connection of the degradative capacity of CTSD [24, 25], CTSB and CTSL [26] with αSyn has been found. In addition, accumulations of αSyn were found in the brains of CTSD-deficient mice [27], which could be diminished after intracranial injection of human recombinant proCTSD, indicating the importance of CTSD in efficient αSyn clearance [25]. On the other side, pathological αSyn can disrupt lysosomal hydrolase trafficking and dramatically decrease the activity of lysosomal hydrolyses as extensively described for GCase [28, 29]. This has a large impact on αSyn levels since decreased GCase activity results in increased levels of its substrate glucosylceramide, which facilitate αSyn aggregation and pathology [30, 31]. Lysosomal cathepsins reach the lysosome mainly via the secretory pathway, where they are processed and mature before reaching the lysosomal structures (as reviewed in [23]). Hence, since lysosomal CTSD, CTSB, and CTSL are directly involved in the degradation of αSyn, the impaired trafficking of those enzymes to the lysosome may contribute to a vicious cycle that accelerates αSyn aggregation and consequently diminishes lysosomal function. In the study of Mazzulli et al., it was shown that the enhancement of hydrolase trafficking by directly targeting vesicular trafficking [32] enhances the activity of GCase. Moreover, activating the lysosomal stress response key player synaptobrevin-2 homolog ykt6, which is a small soluble N-ethylmaleimide-sensitive-factor attachment protein receptor (SNARE) protein that is involved in numerous membrane fusion processes such as endoplasmic reticulum (ER)–Golgi trafficking [33], intra-Golgi transport [34] as well as transport from the recycling endosome to the trans-Golgi network [35], seems to be another potent treatment option to rescue lysosomal function and the activity of the lysosomal enzyme GCase [36–38].

In this study, we utilized an αSyn-overexpressing human neuroglioma cell line (H4) and DA neurons generated from induced pluripotent stem cells (iPSC) of PD patients (*SNCA* triplication and A53T *SNCA* point mutation) to examine the trafficking, maturation and proteolytic activity of CTSD, CTSB, and CTSL as well as therapeutic mechanisms to rescue their enzymatic function. Furthermore, brain samples from mice that overexpress the A53T αSyn mutation in DA neurons were analysed to support our hypothesis in vivo.

## Materials and methods

### Cell culture

#### Human H4 neuroglioma cell culture

Human neuroglioma H4 cell line expressing wildtype αSyn under the control of a tetracycline-inducible promoter (tet-off) was originally established and provided by Pamela McLean (Mayo Clinic, Jacksonville, FL) and has been previously described in Mazzulli et al., 2011 [28]. Cells were cultured in OptiMEM media (Thermo Fisher Scientific, Waltham, MA; #31985070) containing 5% fetal calf serum (FCS) (tet-free; PAN-Biotech, Aidenbach, Germany; #P30-3602), 200 µg/ml geneticin (Thermo Fisher Scientific; #10131035) and hygromycin (Thermo Fisher Scientific; #10687010), and 1% penicillin–streptomycin (Sigma, St. Louis, MO; #P0781). The expression of αSyn was turned off by the addition of 2 µg/ml doxycycline (DOX) (Sigma; # D3447) for 24–72 h. Cells were frequently tested for mycoplasma contaminations (once a month).

#### iPSC culture and neuronal differentiation

PD patient-derived human iPSCs expressing A53T αSyn and isogenic corrected lines were generously provided by Dr. R. Jaenisch (Whitehead Institute MIT) and were extensively described in Soldner et al., 2012 and Cuddy et al., 2019 [36, 39]. Human PD patient-derived iPSCs with an αSyn triplication (3 × *SNCA*), associated isogenic control (iso ctrl) and age-matched healthy control (ctrl) have been previously described [38]. iPSCs were maintained on Matrigel (Corning, Corning, NY; #354234)-coated dishes with mTeSR1 Plus media (Stemcell Technologies, Cologne, Germany; #100–0276) and passed once per week.

The pluripotency of iPSCs was confirmed by immunofluorescence staining of the stem cell markers Nanog, Oct4, Tra-1–60, SSEA4, and SOX2 according to the immunofluorescence protocol described in “Biochemical analyses” section (for detailed information on the antibodies and dilutions, see Table 3). iPSCs were differentiated into midbrain dopaminergic neurons (DA-iPSn) by using an established protocol described previously [40]. In brief, iPSC colonies were enzymatically dissociated by Accutase (Corning, #25–085-Cl) and seeded onto Matrigel-coated 12-well dishes. When reaching 80% confluency, the differentiation protocol was initiated by adding KSR media with dual SMAD inhibitors and carried out for 15 days with the addition of growth and differentiation factors as described earlier [40]. Between days 10 and 15, cell layers were mechanically dissociated into small squares of approximately 2 mm^2^ and plated onto a 6-well dish, which was coated with poly-*d*-lysine (PDL, 33 µg/ml, Merck, #P1149) and 5 µg/ml laminin (Merck, #11243217001). After 25–30 days, the cells were passaged by Accutase (Corning, #25–085-Cl), counted, and plated at a cell number of 8 × 10^4^ cells for activity assay on PDL/laminin-coated 96-well plates, 4 × 10^5^ cells for Western blot analysis on PDL/laminin-coated 24-well plates and 3 × 10^5^ cells for immunofluorescence on PDL/laminin-coated 12-mm coverglasses in a 24-well plate. The growth factors were withdrawn on days 40–50 and aged until day > 90. The iPSC-derived DA neurons were maintained in neurobasal medium (Thermo Fisher Scientific, #21103–049) containing NeuroCult SM1 supplement (StemCell Technologies, #05711), 1% *L*-Glutamin (200 mM stock, Gibco, Billings, MT; #25030–081) and 1% penicillin–streptomycin (Sigma, #P0781). Cells were tested monthly for mycoplasma contaminations.

In order to determine the efficiency of DA-iPSn differentiation, immunofluorescence staining was performed for the neuronal marker beta-III tubulin (TUBB3), and for markers of DA neurons, FOXA2 and tyrosine hydroxylase (TH). Furthermore, the ratios of FOXA2-positive 3 × *SNCA* and isogenic control cells were determined via flow cytometry. The protocols of the immunofluorescence staining and flow cytometry are described in the “Biochemical analyses” section.

### Biochemical analyses

#### Sequential protein extraction of αSyn

Fractionation of soluble and insoluble αSyn was performed as described recently [41]. H4 neuroglioma cells and DA-iPSn were harvested in PBS at 400*g*. Cell pellets and frozen mouse brain tissue were lysed in Triton base buffer (1% Triton X-100, 10% glycerol, 150 mM NaCl, 25 mM HEPES, 1 mM EDTA, 1.5 mM MgCl_2_, pH 7.4) containing 1 × protease inhibitor cocktail (PIC) (cOmplete PIC, Roche, Basel, Switzerland; #11836145001), 50 mM NaF, 2 mM Na_3_VO_4_ and 0.5 mM PMSF. DA-iPSn and brain tissue samples were additionally homogenized with a Homogenizer (Glas-Col model #099C K54, 333–4000 rpm) or a Teflon pestle. Samples were incubated on ice-water slurry for 30 min, frozen and thawed three times, and ultracentrifuged at 100,000 × *g* at 4 °C for 30 min. Supernatant was used as Triton-soluble fraction whereas the remaining pellet was further extracted in SDS base buffer (2% SDS, 50 mM Tris at pH 7.4) containing 1 × PIC. The Triton-insoluble/SDS-soluble fraction was boiled at 99 °C for 10 min, sonicated three times and subsequently ultracentrifuged at 100,000 × *g* at 22 °C for 30 min. Protein concentrations of both fractions were determined by the bicinchoninic acid (BCA) assay (Thermo Fisher Scientific, #23225). Lysates were spiked with 5 × Laemmli containing 0.3 M Tris/HCl pH 6.8, 50% glycerol, 1% SDS, 0.05% bromophenol blue and freshly added 5% 2-Mercaptoethanol.

#### EndoH and PNGaseF treatment

To study the subcellular localization and transport of the cathepsins under different conditions, deglycosylation assays utilizing EndoH and PNGaseF enzymes were performed according to the manufacturer’s handbook (New England Biolabs, Ipswich, MA; #P0702S, #P0704S). Briefly, for both digests, 20 μg of total protein were used, incubated in the provided denaturation buffer and treated with 1 μl of the respective enzyme at 37 °C for 1 h in digestion buffer. Removal of N-linked oligosaccharides was confirmed by Western blot analysis as cleavage of oligosaccharides results in a shift in molecular size of analysed cathepsins. As the EndoH glycosidase is not able to process more complex oligosaccharide chains being found post-ER, sensitivity to EndoH processing was used as a measurement for ER-residency of the protein and thus a measure for the transport of cathepsins. Treatment with PNGaseF was used to determine the protein backbone, as this enzyme is able to also cleave more complex N-linked oligosaccharides forming post-ER.

#### Western blot analysis

Triton-soluble or insoluble lysates (40 µg per lane for H4 cells, 30 µg for DA-iPSn, and 40 µg for mouse brain) were loaded onto 12% SDS-PAGE gels and separated by electrophoresis run at 120 V. Proteins were then transferred onto immobilon-FL PVDF membranes (Millipore, Darmstadt, Germany; #IPFL00010) at a constant voltage (30 V) for 1 h. The membranes were post-fixed in 0.4% paraformaldehyde (PFA) for 20 min as described earlier [42], and blocked in Intercept blocking buffer (Li-Cor, Lincoln, NE; #927-60001) for 1 h. The membranes were incubated overnight with primary antibodies at 4 °C, washed three times in 0.05% TBS-Tween, and Alexa 680- or IRDye800-conjugated secondary anti-rabbit or anti-mouse antibodies (Alexa 680, Thermo Fisher Scientific, or IRDye800, Li-Cor) were added at 1:10,000 in blocking buffer for 1–2 h. To use the same membrane for the analysis of proteins of similar size which were detected with antibodies derived from the same species, the membranes were incubated in 1 × stripping buffer (5 × PVDF Stripping buffer, #928-40032) for 20 min and subsequently checked for remaining signals. If no signals were visible, the blots were washed as described before and scanned using Amersham Typhoon Biomolecular Imager (GE Lifesciences, Chicago, IL) or Odyssey (Li-Cor Biosciences, Lincoln, NE) imaging system. Protein quantification was done by using the Image StudioLite Software (Version 5.2.5, Li-Cor) where the signal intensity values of each protein band of interest were selected. The protein-specific signal intensity was then determined by subtracting the background signal from the total selection signal. Due to variances in the immunoblotting process, the overall intensity values between replicate experiments were corrected for differences by internal normalization. The individual signals for a protein of interest were normalized to the mean protein-specific intensity across all samples, which was done separately for each protein. Next, protein of interest signal was normalized to their respective loading control (GAPDH, β-actin, TUBB3, or CBB). Details for all primary and secondary antibodies used for Western blot analyses are listed in Table 1. Analysis was performed on Microsoft Excel and GraphPad Prism (San Diego, CA).

**Table 1:** Primary and secondary antibodies used for Western blotting

Antibody	Host species	Company	Catalogue number	Dilution
*Primary antibody*				
αSyn (C-20)	Rabbit	Santa Cruz	sc-7011-R	1:1000
αSyn (Poly)	Rabbit	Proteintech	10842-1-AP	1:1000
αSyn (Syn-1)	Mouse	BD	610787	1:500
αSyn (LB509)	Mouse	Abcam	27.766	1:500
αSyn (Syn-211)	Mouse	Santa Cruz	sc-12767	1:500
CTSB	Goat	R&D systems	AF953	1:500
CTSD	Rabbit	Kindly provided by Prof. Andrej Hasilik, Philipps-University Marburg, Germany	N/A	1:500
CTSD	Mouse	BD	610800	1:1000
CTSL	Goat	R&D systems	AF952	1:500
LAMP1	Mouse	DSHB	H4A3	1:1000
LAMP2	Rabbit	Novus Biologicals	NBP2-67298	1:1000
GAPDH	Rabbit	Cell signaling Technology	14C10	1:1000
TUBB3	Mouse	BioLegend	802001	1:1000
β-actin	Mouse	Sigma	A5441	1:5000
*Secondary antibody*				
Alexa Fluor 680 anti-mouse	Donkey	Thermo Fisher Scientific	A-100038	1:10,000
Alexa Fluor 680 anti-goat	Donkey	Thermo Fisher Scientific	A-21084	1:10,000
IRDye 800 anti-mouse	Donkey	Li-Cor	926-32212	1:10,000
IRDye 680 anti-rabbit	Donkey	Li-Cor	926-68073	1:10,000
IRDye 800 anti-rabbit	Donkey	Li-Cor	926-32213	1:10,000
IRDye 800 anti-goat	Donkey	Li-Cor	926-32214	1:10,000

#### Real-time (RT)-qPCR

Cells (H4 and DA-iPSn) were pelleted in PBS at 300 × *g* for 5 min at 4 °C. Total RNA was extracted using the RNeasy Kit according to the protocol (Qiagen, Venlo, The Netherlands; #75162). RNA concentration was measured with a NanoDrop spectrophotometer and 1000-500 ng of RNA was reverse transcribed into cDNA by using the RevertAid First Strand cDNA Synthesis Kit (Thermo Fisher, #K1621) according to the following protocol: 60 min 42 °C, 10 min 70 °C and stored at 4 °C. The RT-qPCR reactions were carried out by using the Roche UPL-Probe system with 4 ng/µl cDNA template in a final reaction volume of 20 µl. The quantification is represented as fold change of the respective mRNA expression normalized to the mean of the housekeeping genes β-actin and GAPDH calculated by the ΔΔCt method. Results are represented as the mean ± SEM of three biological replicates (*n* = 3) with three technical replicates for each. Details for all primer sequences used for RT-qPCR analyses are listed in Table 2.

**Table 2:** Forward and reverse primers used for probe-based RT-qPCR experiments

Gene name	Forward 5’-3’	Reverse 5’-3’	UPL-Probe #
*β-actin*	ATTGGCAATGAGCGGTTC	CGTGGATGCCACAGGACT	11
*CTSB*	TACCTGGTTTGCATAGATGATTG	TGGAAGCCGGATCCTAGA	44
*CTSD*	CATCTTCTCCTTCTACCTGATGCA	GTCTGTGCCACCCAGCAT	64
*CTSL*	TTGGGTAAATGCTTGGGAGA	AAGGCAGCAAGGATGAGTGT	49
*GAPDH*	AGCCACATCGCTCAGACAC	GCCCAATACGACCAAATCC	60
*SNCA*	AAAGGCCAAGGAGGAGGGAGTT	TCTTTGGTCTTCTCAGCCACTA	66

#### Immunofluorescence staining

iPSC, DA-iPSn and H4 cells were fixed in 4% PFA in PBS for 20 min at room temperature (RT), permeabilized with 0.3% Triton X-100 (Roth, Frederikssund, Denmark; #3051.2) in PBS for 30 min and subsequently blocked in blocking buffer containing 2% BSA and 5% FCS in 0.3% Triton X-100 PBS for 1 h. For lysosomal protein staining, cells were permeabilized with 0.2% saponin (Sigma, #47036-50G-F) in PBS for 5 min, followed by 0.2% saponin together with 0.2% glycine in 1 × PBS for 10 min, and then blocked with 0.2% saponin, 0.2% glycine and 10% FCS in PBS. Primary antibodies were diluted in blocking buffer and incubated overnight at 4 °C. Cells were washed three times in 0.3% Triton X-100 in PBS or 0.2% saponin in PBS, and incubated with secondary antibodies for 1 h at RT. Cells were repeatedly washed three times in 0.3% Triton X-100 in PBS or 0.2% saponin in PBS, and stained with DAPI (Sigma, #13190309) diluted 1:10,000 in PBS, incubated for 10 min at RT. Cells were washed three times with PBS. Images of iPSC marker staining were acquired with EVOS™ 5000 microscope (Invitrogen, Carlsbad, CA). Cells stained for DA-iPSn markers and colocalization experiments were mounted in Prolong Gold antifade reagent (Invitrogen, #P36930) and analysed by a confocal laser scanning microscope (IX83, Olympus) or ZEISS (LSM 780, Carl Zeiss Microscopy GmbH) with digital images processed using Inspector Image Acquisition & Analysis Software (Abberior Instruments) or Zeiss blue software (ZEN lite 2012). Details for all primary and secondary antibodies used for immunofluorescence analyses are listed in Table 3.

**Table 3:** Primary and secondary antibodies used for immunofluorescence analysis

Antibodies used for iPSC characterization				
Primary antibodies	Host species	Company	Catalogue number	Dilution
Oct4	Rabbit	Abcam (Human Embryonic Stem Cell Marker Panel)	ab109884	1:100
Tra-1–60	Mouse			1:100
Sox2	Rabbit			1:100
Nanog	Rabbit			1:100
SSEA4	Mouse			1:100

Secondary antibodies	Host species	Company	Catalogue number	Dilution
AlexaFluor 488 anti-rabbit	Donkey	Thermo Fisher Scientific	A-21206	1:2000
AlexaFluor 568 anti-mouse	Donkey	Thermo Fisher Scientific	A-10037	1:2000

Antibodies used for DA-iPSn characterization				
Primary antibodies	Host species	Company	Catalogue number	Dilution
TH	Rabbit	Millipore	AB5986	1:500
FOXA2	Mouse	Santa Cruz	sc101060	1:400
TUBB3	Mouse	Biolegend	801202	1:1000

Secondary antibodies	Host species	Company	Catalogue number	Dilution
AlexaFluor 488 anti-mouse	Donkey	Thermo Fisher Scientific	A-21202	1:500
AlexaFluor 594 anti-rabbit	Goat	Thermo Fisher Scientific	A-11037	1:500

Antibodies used for lysosomal protein stainings				
Primary antibodies	Host species	Company	Catalogue number	Dilution
CTSB	Goat	R&D systems	AF953	1:100
CTSD	Rabbit	Kindly provided by Prof. Andrej Hasilik, Philipps-University Marburg, Germany	N/A	1:100
LAMP2	Mouse	DSHB	H4B4	1:200

Secondary antibodies	Host species	Company	Catalogue number	Dilution
AlexaFluor 488 anti-mouse	Donkey	Thermo Fisher Scientific	A-21202	1:500
AlexaFluor 596 anti-rabbit	Donkey	Thermo Fisher Scientific	A-21207	1:500
AlexaFluor 647 anti-goat	Donkey	Invitrogen	A-21447	1:500

##### Quantification of co-localisation and lysosomal CTSD and CTSB intensity

The Pearson correlation coefficient was determined by ImageJ/Fiji software. Cells were marked by a region of interest and co-localization of two signals (in different channels) (cathepsins and lysosomal marker protein LAMP2) was determined by the COLOC 2 plugin. Positive values describe a positive correlation (co-localization) of two stainings with “1” being the highest possible value.

To quantify the intensity of CTSD and CTSB within lysosomes, ImageJ/Fiji software was used. First, the scale of the picture was set to 8 pixels per micron, then a region of interest was created around each cell. The vesicles positive for lysosomal localisation of CTSD and CTSB were selected by choosing a colour threshold for co-localisation with LAMP2 (yellow signal; hue setting: 31–53). For each selected vesicle, the “mean” of signal intensity and “area” output values were multiplied to calculate the total signal intensity per micron [8 pixels].

#### Flow cytometry analysis (FACS) of DA-iPSn

For nuclear expression analysis of FOXA2 in 3 × *SNCA* and isogenic control cells, 5 × 10^5^ cells were harvested and subsequently fixed with BD Cytofix (BD Cytofix/Cytoperm™ Fixation/Permeabilization Solution Kit with BD GolgiStop™, 555028, BD Biosciences GmbH). Cells were permeabilized using Cytoperm™ (0.1% Triton X-100 in PBS). Solutions were prepared according to manufacturer’s protocol. For nuclear FOXA2 staining, cells were incubated with the primary antibody (HNF-3β (RY-7), sc-101060, Santa Cruz Biotechnology, Dallas, TX) for 30 min at 4 °C. Prior to secondary antibody staining, cells were washed using Cytoperm™ (0.1% Triton X-100 in PBS). Cells were stained with secondary antibody (donkey anti-mouse IgG (H + L), Alexa Fluor™ 568, A10037, Invitrogen) for 20 min at 4 °C in the dark. Secondary antibody was rinsed as described above. Stained cells were diluted in 500 µl PBS (2% fetal bovine serum) and subsequently analysed with the BD LSRFortessa™ Cell Analyzer (BD Biosciences GmbH, Franklin Lakes, NJ). Proportions of FOXA2 + cells were calculated using FlowJo™ Software (BD Biosciences GmbH).

#### Lysosomal enrichment

For lysosomal enrichment, H4 cells were seeded onto 15-cm dishes (Sarstedt, Nümbrecht, Germany; #83.3903) at a cell number of 2 × 10^6^ per dish. For lysosomal enrichment of DA-iPSn, 4 wells of a 24-well plate (each well containing 3.5 × 10^5^ cells) were combined per sample. Cells were washed and harvested with PBS. After centrifugation at 400 ×* g* for 5 min at 4 °C, PBS was aspirated. 400 µl (200 µl in the case of DA-iPSn) sucrose HEPES buffer (250 mM sucrose [AppliChem, #A2211], 10 mM HEPES, 100 mM EDTA, pH 7.4) was added. Cells were then homogenized utilizing a cell homogenizer (Glas-Col, Terre Haute, IN; #099D GT31) and centrifuged at 6800 × *g* for 5 min at 4 °C to remove unbroken cells and debris. The supernatant containing lysosomes was then collected. The homogenization step of the pellet was repeated and the combined supernatant was centrifuged once more at 17,000 × *g* for 10 min at 4 °C. For Western blot analysis, the lysosome-containing pellet was lysed in Triton base buffer (1% Triton X-100, 10% glycerol, 150 mM NaCl, 25 mM HEPES, 1 mM EDTA, 1.5 mM MgCl_2_, pH 7.4) containing freshly added 1 × PIC (cOmplete PIC, Roche, #11836145001), 50 mM NaF, 2 mM Na_3_VO_4_ and 0.5 mM PMSF. For CTSD activity assay, the pellet was lysed in low-pH Triton buffer (50 mM sodium acetate, 0.1 M NaCl, 1 mM EDTA, and 0.2% Triton X-100; pH 4.5). The lysate was incubated for 10 min on ice-slurry and subsequently centrifuged (20,000 ×* g*, 15 min, 4 °C). Protein concentration was determined via BCA method (Thermo Fisher Scientific, 23225).

#### CTSD, CTSB and CTSL enzymatic activity assays

To measure CTSD activity, 5 µg of cell lysate freshly enriched for lysosomes or mouse brain lysate was incubated in 100 µl lysis buffer (50 mM sodium acetate, 0.1 M NaCl, 1 mM EDTA, 0.2% Triton X-100) containing 10 µM quenched fluorogenic peptide (Enzo, New York, NY; #BML-P145) and 25 µM leupeptin (Enzo, #ALX-260-009-M025) at 37 °C for 30 min. The addition of CTSD inhibitor pepstatin A (PepA; Sigma-Aldrich, St. Louis, MO; #P5318) was used as a negative control. Fluorescence signal was measured for each sample in triplicates with a plate reader (SpectraMax Gemini, Molecular Devices, San José, CA, excitation: 322 nm; emission: 381 nm). The determination of CTSB and CTSL enzymatic activities was done under the same conditions, utilizing 20 µM quenched fluorogenic peptide (Enzo, #BML-P139-0010) for CTSB and 9.4 µM (BioRad; #ICT942) of fluorescent probe for CTSL. Fluorescence signals were measured at excitation: 365 nm and emission: 440 nm for CTSB, and excitation: 590 nm and emission: 628 nm for CTSL. All values were corrected for background fluorescence.

#### Live-cell lysosomal enzyme activity assays

Enzymatic activities of CTSB and CTSL were assessed in living cells. H4 cells and iPSn were seeded on a dark 96-well plate with clear bottom (Thermo Fisher Scientific, #265301) and maintained in culture media until analysis. Cells were loaded with cascade blue dextran at a concentration of 1 mg/ml (Thermo Fisher Scientific, #D-1976) 24 h before measurement. On the day of experiment, cells were treated with either 200 nM bafilomycin A1 (BafA1) (Santa Cruz, #sc-201550A) or DMSO for 1 h. Cascade blue dextran was then washed out, and cells were pulse chased with cell-permeable substrates MagicRed (RR_2_) (BioRad; #ICT938) for CTSB activity and MagicRed (FR_2_) (BioRad; #ICT942) for CTSL activity as indicated in the manual for 1 h. After substrate incubation, cell medium was replaced by phenol red-free Optimem (Thermo Fisher Scientific, #11058021) for H4 cells and neurobasal medium (Thermo Fisher Scientific, #12348017) and fluorescence intensity was recorded every 30 min for 3 h in a plate reader (Gemini EM, Molecular Devices). MagicRed substrates were measured at excitation of 592 nm and emission of 628 nm and cascade blue dextran at excitation: 400 nm and emission: 430 nm. After the last read, cells were fixed in 4% PFA in PBS, washed with PBS and permeabilized with 0.3% Triton X-100 for 30 min at RT. Cells were blocked in Intercept blocking buffer for 1 h and stained with celltag700 (LiCor; # 926-41090) for another 1 h. Cells were then washed three times and scanned with Amersham Typhoon Biomolecular Imager infrared imaging system. Fluorescence intensities of enzymatic substrates were normalized to lysosomal volume (cascade blue dextran) or cell volume (celltag700), graphed as fluorescence intensity vs time, and analysed by using the area under the DMSO and BafA1 curve (AUC). Lysosomal activity was obtained by substracting the AUC of BafA1 from DMSO curves. Substracted AUC values are shown in bar graphs with ± SEM.

### Farnesyltransferase inhibitor (FTI) treatment of cell cultures and mice

#### Treatment of cultures

H4 cells or DA-iPSn were treated with vehicle (DMSO) and 5 nM or 10 nM FTI (LNK-754). H4 cells were treated for 5 days and medium was changed every day. DA-iPSn were treated for 7 days and medium was changed every other day for the time of the experiment. CTSD:LAMP2 and CTSB:LAMP2 colocalization was determined via immunofluorescence analysis and CTSD activity was measured after lysosomal enrichment by a CTSD enzyme activity assay. CTSB and CTSL activity was measured by a live-cell lysosomal activity assay. Treatment of H4 cells with PepA (Millipore, #5.08437.0001) and E64 (Thermo Fisher Scientific, #78434) was conducted for 5 days, with medium change every day. DA-iPSn were treated for 14 days, with medium change every second day. Sequential protein extraction was performed for subsequent Western blot analyses.

#### Treatment of mice with FTI

Mouse samples were derived from animals treated and handled as described in Cuddy et al., 2019 [36]. Mice were bred and housed according to the Institutional Animal Care and Use Committee at Northwestern University guides and handled in accordance with the US National Institutes of Health Guide to the Care and Use of Laboratory Animals and Society for Neuroscience guidelines. Mice had access to water ad libitum and were provided with standard rodent chow. The genotyping was performed using tail clipping samples by Transnetyx (https://www.transnetyx.com). The use of the animals was approved under the Northwestern IACUC protocol number IS00011551.

LNK-754 was formulated in a vehicle of 0.5% sodium carboxymethylcellulose and filtered before use. DA_SYN53_ mice were intraperitoneally (i.p.) injected daily with 0.9 mg/kg LNK-754 for 26 days. Following the end of the FTI treatment, the animals were perfused with PBS and the midbrain/thalamic brain region (from a 1-mm thick coronal section, corresponding to bregma −2.5 to 3.5 mm) was rapidly dissected and frozen until further biochemical analysis. The study groups had an identical male-to-female ratio, and the animals used for the experiments were 9–14 months old.

### Statistical analysis

For statistical analysis, GraphPad Prism version 9 (Graph Pad Software, Inc., San Diego, CA) was used. Data were analysed with one-way analysis of variance (ANOVA) followed by Dunnett’s or Tukey’s *post-hoc* test for multiple comparisons or with a two-tailed Student’s *t*-test for comparisons between two groups. Data are expressed as mean ± SEM. *P* < 0.05 was considered statistically significant. Blinding and randomization were performed whenever possible. The distribution of the data was assumed to be normal, but this was not formally tested.

## Results

### High αSyn levels impair maturation, activity and trafficking of cathepsins to the lysosome in H4 cells

**Fig. 1:**
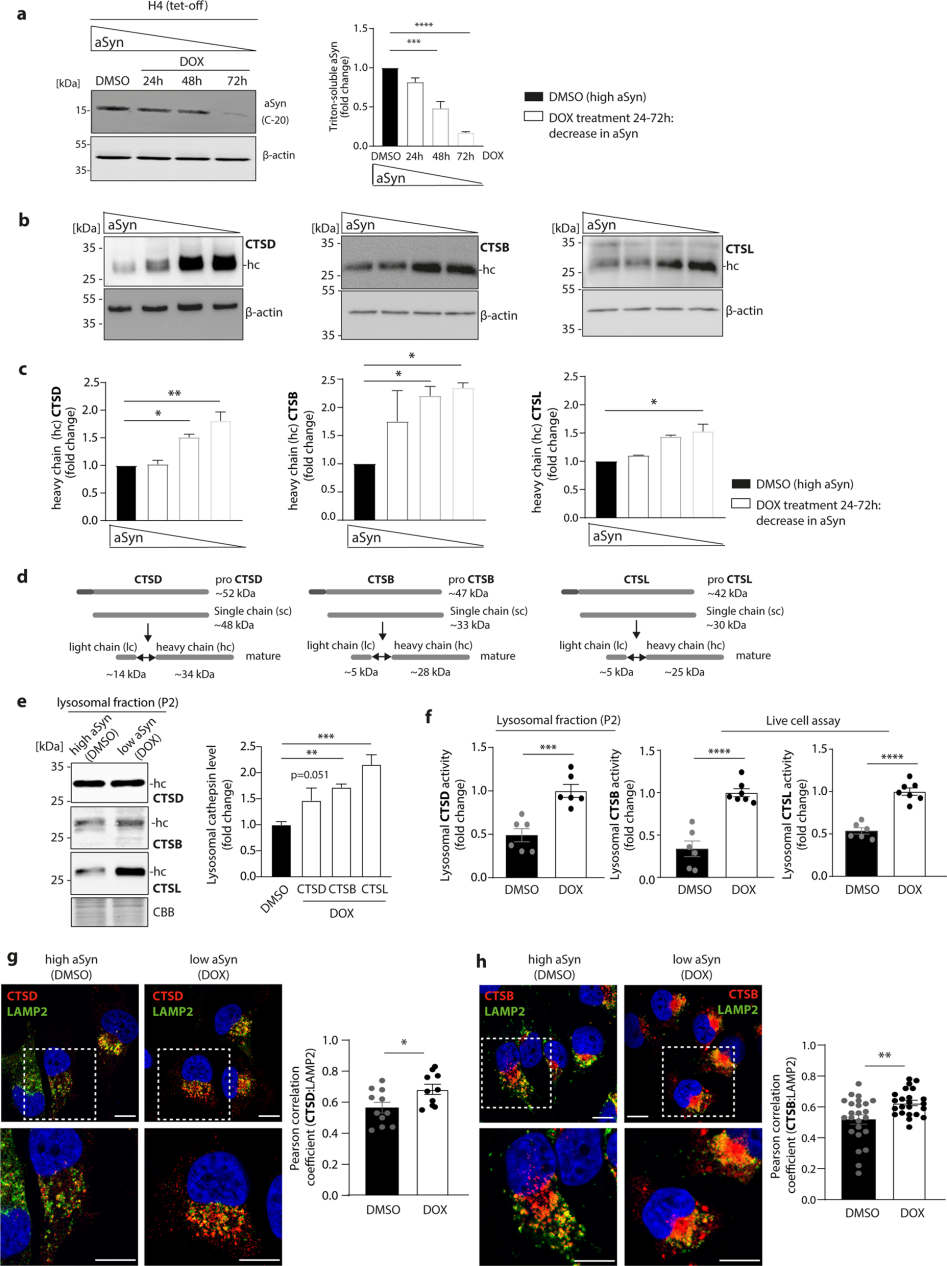
Disrupted trafficking and activity of cathepsins in H4 cells overexpressing αSyn. **a** Representative Western blot analysis of αSyn in H4 cells overexpressing αSyn under an inducible tetracycline (tet)-off promoter. The addition of doxycycline (DOX) for 24 h, 48 h, and 72 h results in down-regulation of αSyn protein levels compared to control samples (cells treated with DMSO for 72 h). For loading control β-actin staining was performed (*n* = 3). **b** Representative Western blot analyses of the heavy chain (hc) representing the mature form of cathepsin D (CTSD), cathepsin B (CTSB) and cathepsin L (CTSL) in H4 cells overexpressing αSyn under an inducible tetracycline (tet)-off promoter. αSyn expression was downregulated by the addition of DOX for 24 h, 48 h and 72 h. Control cells were treated with the same amount of the DOX dissolvent DMSO (high αSyn) for 72 h. As a loading control β-actin was stained to ensure equal protein load. **c** Corresponding quantification of Western blot analysis for CTSD (left), CTSB (middle) and CTSL (right). Cells were treated with DMSO for 72 h or with DOX for 24, 48, or 72 h and were normalized to β-actin and expressed as fold change, compared to high αSyn levels (DMSO) (*n* = 3). **d** Schematic illustration of the maturation process of CTSD, CTSB, and CTSL. The inactive pro-forms of all three enzymes are generated in the endoplasmatic reticulum (ER). The pro-forms are transported into endosomes, where they are cleaved, resulting in the formation of single chains (sc) of the enzymes. The final maturation step takes places in the lysosomes, where a further cleavage of the sc leads to the production of a heavy and a light chain (hc and lc, respectively). **e** Western blot analysis of hc of CTSD, CTSB and CTSL in lysosome-enriched fractions of DMSO- and DOX (for 72 h)-treated H4 cells. All three investigated cathepsins show increased levels upon αSyn expression downregulation (cell treated with DOX) (*n* = 2–6). **f** Lysosomal CTSD, CTSL, and CTSB activity in H4 cells high in αSyn (DMSO) compared to low αSyn (treated with DOX for 72 h). Lysosomal CTSD activity was assessed by measuring activity within fractions enriched for lysosomes. Lysosomal CTSB and CTSL were determined by live-cell activity assay (*n* = 6–7). **g** Left: Representative immunofluorescence images of CTSD (red) co-stained with lysosomal protein LAMP2 (green) in H4 cells high in αSyn (DMSO) and low in αSyn (DOX). The nucleus is shown in blue and stained with DAPI. Scale bar: 10 µm. Right: Quantification of CTSD:LAMP2 co-staining determined by Pearson correlation coefficient (*n* = 11 individual cells per group). **h** Left: Representative immunofluorescence images of CTSB (red) co-stained with lysosomal protein LAMP2 (green) in H4 cells high in αSyn (DMSO) and low in αSyn (DOX). The nucleus is shown in blue and stained with DAPI. Scale bar: 10 µm. Right: Quantification of CTSB:LAMP2 co-staining determined by Pearson correlation coefficient (*n* = 23–24 individual cells per group). Statistical analyses were performed by using one-way ANOVA together with Dunnett’s multiple comparison test for **a**, **c**, and **e**, and two-tailed unpaired Student’s *t*-tests for **f**–**h**. ****P < 0.0001, ***P < 0.001, **P < 0.01, *P < 0.05

Since earlier studies indicate that aggregated αSyn is able to disrupt the degradative capacity of lysosomes by interfering with proper hydrolyse trafficking [28, 32], we first examined the levels of mature forms of CTSD, CTSB, and CTSL in a human neuroglioma cell line (H4) by Western blot analysis. This H4 cell line stably overexpresses αSyn under a tetracycline (tet)-responsive promoter in a tet-off manner, meaning that αSyn expression can be gradually downregulated on mRNA and protein levels by adding DOX (a second-generation tetracycline) to the cell culture media for 24 h, 48 h, and 72 h (Fig. 1a and Additional file 1: Fig. S1a). Treatment with DMSO for 72 h retained αSyn expression and thus high intracellular αSyn level (Fig. 1a and Additional file 1: Fig. S1a). The signal intensity of the heavy chain of all three cathepsins (CTSD ~ 34 kDa; CTSB ~ 28 kD and CTSL ~ 25 kDa) increased gradually when αSyn levels were diminished (Fig. 1b). Quantification of the signal intensities showed significant increases in the levels of heavy chain of CTSD, CTSB and CTSL following 24–72-h DOX treatment (Fig. 1c).

To investigate the effects of αSyn levels on the different forms of cathepsins produced during their maturation [23] (Fig. 1d), we analysed the levels of pro- and single-chain forms as well as the mature form (heavy chain) of CTSD in DMSO- and DOX-treated H4 cells. As cleavage of the pro-peptide is mediated after leaving the Golgi apparatus within endosomes and further maturation resulting in a light chain and a heavy chain is mediated under lysosomal pH, the different forms can be used to evaluate lysosomal cathepsin trafficking [23]. Our results showed that while the levels of mature CTSD (heavy chain) increased upon decreasing αSyn levels, the pro-form of the protein diminished, suggesting enhanced maturation and intracellular trafficking of the enzyme (Additional file 1: Fig. S1b, d). Utilizing an EndoH digest that is only capable of processing N-linked oligosaccharide side chains as being present in the ER, we measured ER-residency of CTSD. Under low αSyn conditions (DOX treatment), less CTSD EndoH sensitivity was observed, indicating further post-ER trafficking and maturation of the lysosomal enzyme (Additional file 1: Fig. S1c, d).

As cathepsins fulfil their protease function in the lysosomes, we next examined the effect of αSyn levels on the amount of mature CTSD, CTSB, and CTSL levels in lysosomal structures. For this, we performed lysosome enrichment of DMSO- and DOX-treated H4 cells by sequential centrifugation (as applied in [25] and [43]), and performed Western blot analysis with the LAMP1-enriched P2 lysosome-enriched fractions (Additional file 1: Fig. S1e). Analysis of cathepsin levels showed increases in the amount of the heavy chains of all three enzymes under low αSyn (DOX) in comparison to high αSyn (DMSO) condition (Fig. 1e).

Next, the proteolytic activity of the analysed cathepsins was determined by two different approaches. To analyse CTSD activity, a fluorogenic assay was used in lysosome-enriched fractions. CTSB and CTSL activities were determined by a live-cell approach capable of distinguishing lysosomal vs. non-lysosomal activity [44]. Lysosomal proteolytic activity of all three cathepsins was significantly diminished in H4 cells overexpressing αSyn (DMSO) (Fig. 1f). To exclude changes in proteolytic activity due to variations in gene transcription, mRNA levels of CTSD, CTSB and CTSL were assessed by RT-qPCR analysis and found to be unaltered by DOX treatment up to 72 h (Additional file 1: Fig. S1a). Since enzymetic activity was declined in cells overexpressing αSyn, we speculate that the transport of cathepsins to their destination might be disturbed. Immunofluorescence co-staining for CTSD or CTSB with lysosomal marker LAMP2 revealed a higher colocalization value in the H4 cells with low αSyn level (DOX for 72 h), compared to the H4 cells with high αSyn level (DMSO) (Fig. 1g, h). These results favour the hypothesis that the transport of cathepsins to the lysosome is dysfunctional, affecting the proteolytic activity inside the lysosome.

### Impaired lysosomal trafficking of CTSD, CTSB and CTSL in PD-derived DA-iPSn

To determine whether these observations in H4 cells can be replicated in a more physiologically relevant cell model, we used iPSCs derived from PD patients harbouring a genetic *SNCA* triplication (3 × *SNCA*) [38] or αSyn A53T point mutation [39]. The pluripotency of iPSCs was confirmed by immunofluorescence staining of the stem cell markers Nanog, Oct4, Tra-1–60, SSEA4, and SOX2 (Additional file 1: Fig. S2a–d). iPSCs were further differentiated into midbrain DA neurons [40] and cultivated for > 90 days before analysis (referred to as “DA-iPSn”).

Both lines (3 × *SNCA* and A53T) as well as respective isogenic control were analysed for the differentiation efficiency towards DA neurons via immunofluorescence using antibodies against the DA neuron markers TH and transcription factor FOXA2, as well as the neuronal marker TUBB3. A percentage of TH/FOXA2 double-positive cells above 80% in all analysed lines indicates efficient differentiation towards DA neurons (Additional file 1: Fig. S3a–d), which is further confirmed by FACS analysis using FOXA2 (Additional file 1: Fig. S3a, b).

**Fig. 2:**
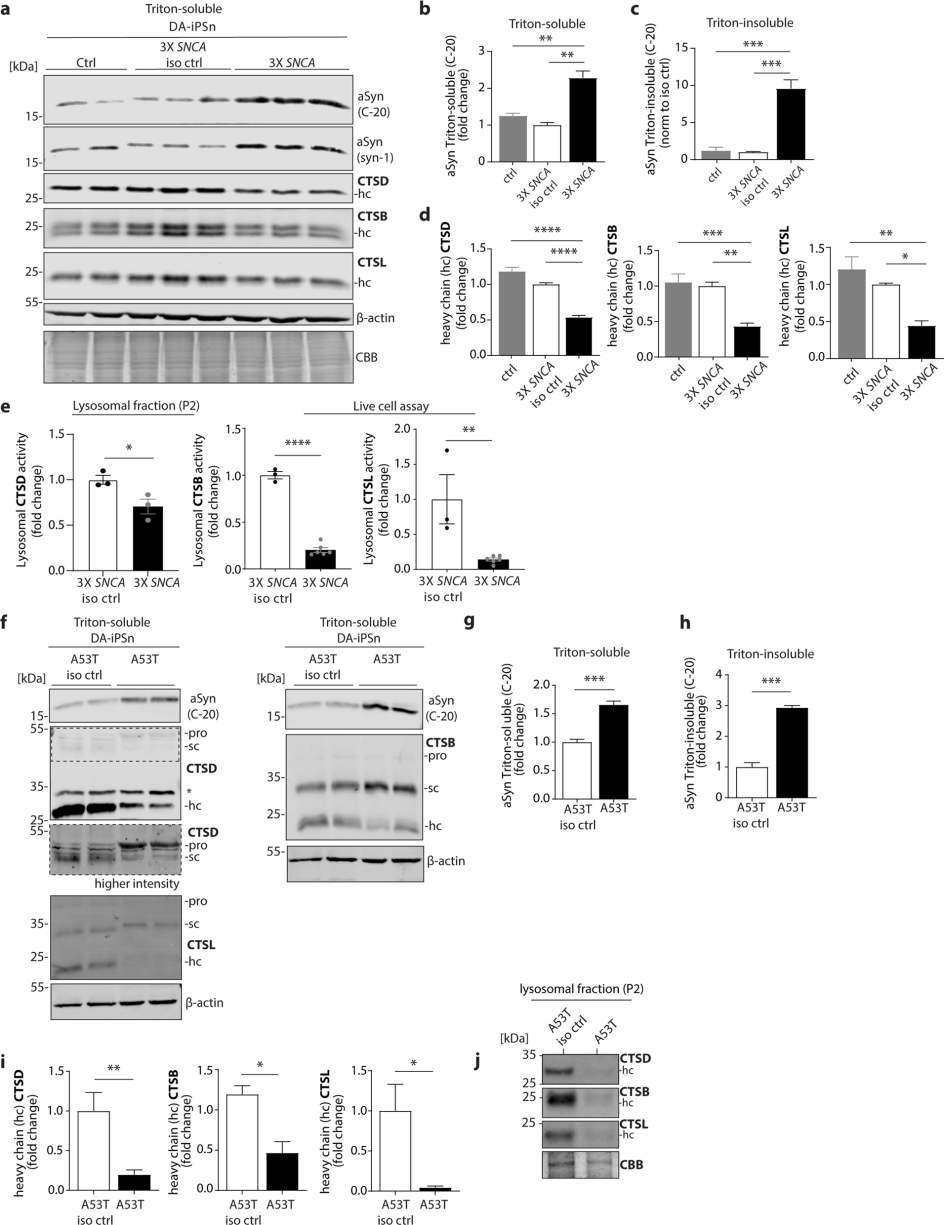
Impaired maturation and proteolytic activity of cathepsins in iPSC-derived DA midbrain neurons with pathological αSyn levels. **a** Representative Western blot analysis of Triton-soluble lysates of DA-iPSn of healthy control (ctrl), isogenic control (iso ctrl) and 3 × *SNCA* showing mature forms (heavy chain; hc) of CTSD, CTSB, and CTSL as well as αSyn. β-Actin served as a loading control. **b** Quantification of Triton-soluble αSyn signals (with C-20 antibody) in DA-iPSn iso ctrl, ctrl and 3 × *SNCA*. Signals were normalized to β-actin and expressed as fold change, compared to 3 × *SNCA* iso ctrl (*n* = 3–6). **c** Quantification of Triton-insoluble αSyn signals (with C-20 antibody) in DA-iPSn iso ctrl, ctrl and 3 × *SNCA*. Signals were normalized to β-actin and expressed as fold change, normalized to 3 × *SNCA* iso ctrl (*n* = 3). For the analysed Western blot image please see Additional file 1: Fig. S4a. **d** Quantifications of the hc of CTSD (left), CTSB (middle) and CTSL (right) in DA-iPSn iso ctrl, ctrl and 3 × *SNCA*. Each signal was normalized to the corresponding β-actin signal and shown as fold change, compared to iso ctrl (*n* = 3–6). **e** Lysosomal CTSD activity conducted from lysosome-enriched fractions (P2), and CTSL and CTSB activity assessed by live-cell activity assay in DA-iPSn iso ctrl, ctrl and 3 × *SNCA* (*n* = 3–5). **f** Representative Western blot analysis of αSyn levels and pro-form/single chain (sc) and heavy chain (hc) of cathepsins in A53T *SNCA* mutant DA-iSPn compared to the iso ctrl. β-actin signals were used to ensure equal protein loading. The asterisk (*) next to the CTSD blot marks an unspecific band. **g** Quantification of Triton-soluble αSyn (detected with C-20 antibody) in A53T neurons compared to iso ctrl. Signal intensity was normalized to β-actin signal and displayed as fold change (*n* = 4). **h** Quantification of Triton-insoluble αSyn (with C-20 antibody) in A53T neurons compared to iso ctrl. Signal intensity was normalized to β-actin signal and displayed as fold change (*n* = 3). For the analysed Western blot image please see Additional file 1: Fig. S4f. **i** Quantifications of the hc of CTSD (left), CTSB (middle), and CTSL (right) normalized to β-actin and expressed as fold change, compared to A53T iso ctrl (*n* = 4). **j** Western blot analysis of hc of CTSD, CTSB and CTSL in lysosome-enriched fractions of A53T and iso ctrl neurons. All three investigated cathepsins show decreased levels in A53T mutant cells compared to the iso ctrl. Statistical analyses were performed by using one-way ANOVA together with Tukey’s multiple comparison test for **b–d**, and two-tailed unpaired Student’s *t*-tests for **e–i** ****P < 0.0001, ***P < 0.001, **P < 0.01, *P < 0.05

The DA-iPSn derived from PD patients harbouring 3 × *SNCA* demonstrated a significant increase of soluble αSyn levels in comparison to a healthy age-matched control (Ctrl) as well as the respective isogenic control (Fig. 2a, b). Insoluble αSyn was also clearly detected in the Triton-insoluble (SDS-soluble) fraction of 3 × *SNCA* neurons by using two different αSyn antibodies: the pathology-related LB509 and non-pathology-related C-20 antibody (Fig. 2c; Additional file 1: Fig. S4a, b). Further, the signal intensities of the mature forms (heavy chain) of CTSD, CTSB and CTSL in DA-iPSn 3 × *SNCA* revealed a significant decrease in comparison to the corresponding controls (Fig. 2a, d; Additional file 1: Fig. S4c, d).

Furthermore, the proteolytic activity of the three cathepsins CTSD, CTSB, and CTSL was evaluated in DA-iPSn 3 × *SNCA* in comparison to the isogenic control (Fig. 2e). For CTSD, activity was determined in lysosome-enriched P2 fractions of the samples. The P2 fractions showed increased signal of the lysosomal marker LAMP2, and decreased signal of GAPDH (Additional file 1: Fig. S4e). Lysosomal CTSB and CTSL were assessed via a live-cell activity assay that is capable of distinguishing lysosomal from non-lysosomal enzyme activity [44]. Both assays showed compromised lysosomal activity of all analysed cathepsins in DA-iPSn harbouring synuclein aggregation (3 × *SNCA*) in comparison to the isogenic controls (Fig. 2e). The A53T mutation in the *SNCA* gene is known to result in increased αSyn protein level and consequently accelerates αSyn aggregation [3]. In line with this, an increase in αSyn protein level was verified by Western blot analysis (Fig. 2f, g). Insoluble αSyn conformers were also clearly detected in the Triton-insoluble fraction of A53T neurons by using the pathology-related LB509 [45] and non-pathogenic αSyn-related C-20 antibody (Fig. 2h; Additional file 1: Fig. S4f, g).

Western blot analysis showed reduced signal intensities of CTSD, CTSB and CTSL heavy chains in A53T DA-iPSn compared to the isogenic control cells both in whole-cell lysates (Fig. 2f, i) and in the lysosome-enriched P2 fractions (Fig. 2j).

RT-qPCR showed a significant increase in *SNCA* mRNA level in DA-iPSn harbouring the αSyn triplication (3 × *SNCA*) as expected. However, no significant changes were seen for the analysed cathepsins when comparing patient neurons to their respective controls (Additional file 1: Fig. S4h, i). These data indicate a disturbance of lysosomal activity and maturation of CTSD, CTSB, and CTSL in two different DA-iPSn models of PD harbouring synucleinopathy, which may further drive αSyn pathology.

### Boosting lysosomal transport reduces αSyn and improves trafficking of cathepsins in H4 cells overexpressing αSyn

**Fig. 3:**
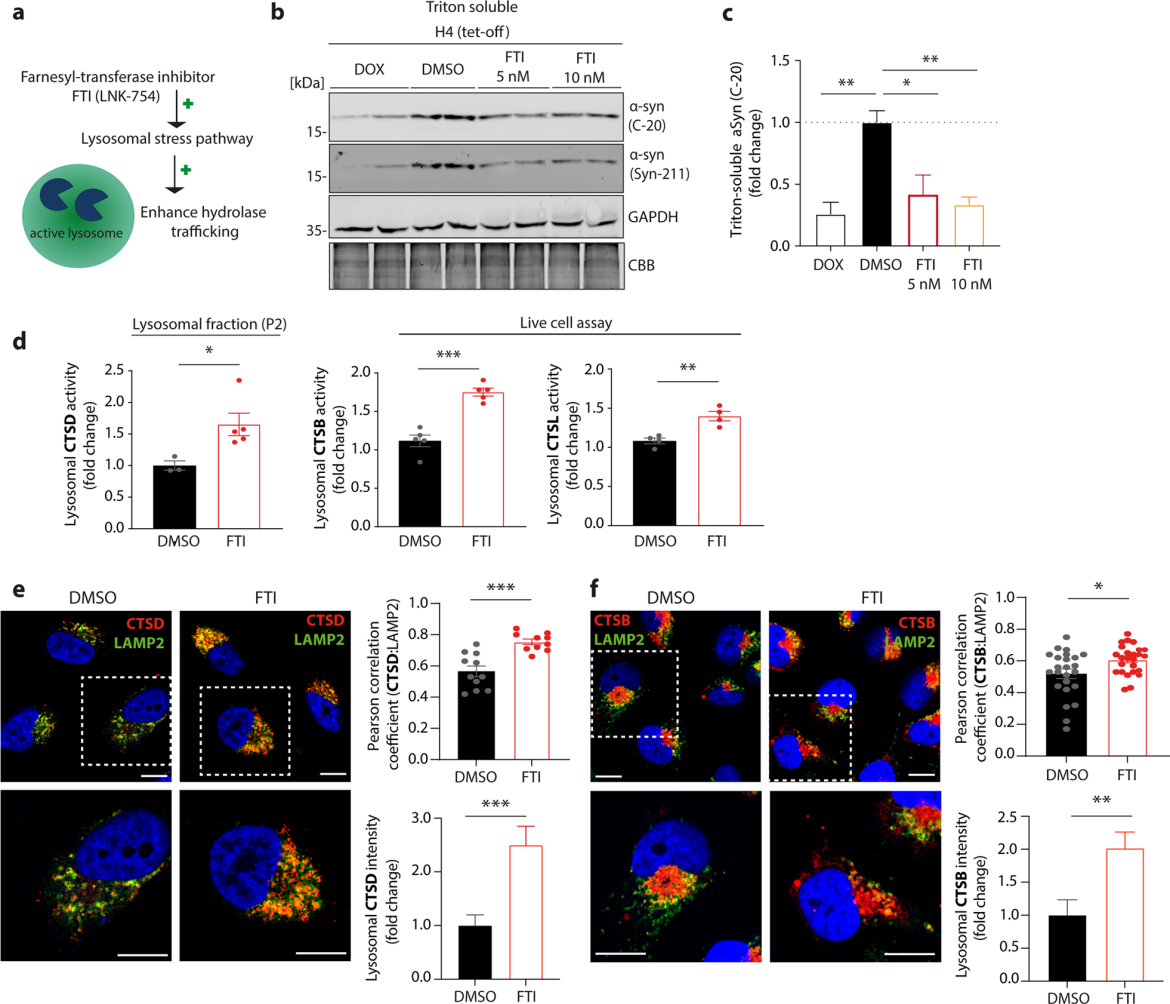
Farnesyltransferase inhibitor (FTI) treatment decreases αSyn level and ameliorates impaired cathepsins trafficking in H4 cells overexpressing αSyn. **a** Schematic illustration of FTI (LNK-754) mechanism inside the cell. The application of the compound induces a lysosomal stress pathway, which boosts hydrolase trafficking towards the lysosome. **b** Western blot analysis of αSyn using C-20 and Syn-211 antibodies in H4 cells cultured with 5 nM or 10 nM FTI for 5 days with every day medium change. DOX treatment was used as a positive control to show low αSyn levels. GAPDH and CBB served as loading controls. **c** Corresponding quantification of αSyn signals detected with C-20 αSyn antibody in H4 cells, which were normalized to GAPDH and shown as fold change, compared to high αSyn (DMSO) (*n* = 4–5). **d** Lysosomal CTSD activity of lysosome-enriched fraction and live-cell lysosomal CTSL and CTSB activity assay of H4 cells overexpressing αSyn (DMSO), with and without FTI treatment (5 days treatment with every day medium change, *n* = 3–5). **e** Left: Representative immunofluorescence images of αSyn-overexpressing (DMSO-treated) H4 cells with or without 5 nM FTI treatment, stained for CTSD (red) and co-stained with LAMP2 (green). Nucleus is stained with DAPI. Scale bars: 10 µm. Right: Pearson correlation coefficient analysis of CTSD:LAMP2 co-staining, and lysosomal CTSD intensity analysis in H4 cells with or without 5 nM FTI treatment (Pearson correlation coefficient: *n* = 10–11 individual cells per group; lysosomal CTSD intensity analysis: *n* = 10–12 individual cells per group). **f** Left: representative immunofluorescence images of αSyn-overexpressing (DMSO-treated) H4 cells with or without 5 nM FTI treatment, stained for CTSB (red) and co-stained with LAMP2 (green). Nucleus is stained with DAPI. Scale bars: 10 µm. Right: Pearson correlation coefficient analysis of CTSB:LAMP2 co-staining, and lysosomal CTSB intensity analysis in H4 cells with or without 5 nM FTI treatment (Pearson correlation coefficient: *n* = 24–26 individual cells per group; lysosomal CTSB intensity analysis: *n* = 10 individual cells per group). Statistical analyses were performed by using one-way ANOVA together with Dunnett’s post-hoc test for **c**, and two-tailed unpaired Student’s *t*-tests for **d–f**. ****P < 0.0001, ***P < 0.001, **P < 0.01, *P < 0.05

Since our previous data utilizing H4 cells and DA-iPSn suggest an αSyn-dependent disturbance of CTSD, CTSB, and CTSL maturation and lysosomal trafficking, we used a small compound (LNK-754) that had recently been shown to increase lysosomal protein trafficking via the secretory pathway [36–38]. LNK-754 is a FTI known to activate ykt6, a SNARE protein involved in lysosomal stress response that has been shown to mediate hydrolase trafficking to the lysosome [36] (Fig. 3a). H4 cells exhibiting low (DOX) and high αSyn levels (DMSO) were treated with 5 or 10 nM FTI for 5 days and tested for restoration of lysosomal maturation and proteolytic activity of CTSD, CTSB, and CTSL. Western blot analysis of H4 cells treated with FTI replicated the reduction of αSyn levels as described previously [36] (Fig. 3b). Quantification of the αSyn level after FTI treatment verified the reduction of αSyn to similar levels as that in the cells treated with DOX for 72 h (Fig. 3c). The levels of insoluble αSyn conformers detected with the pathology-related LB509 antibody were also decreased upon FTI treatment in Triton-insoluble fractions of αSyn-overexpressing H4 cells (Additional file 1: Fig. S5a, b). As both 5 and 10 nM FTI treatments resulted in similar, significant decreases of soluble and insoluble αSyn, the lower concentration (5 nM) was used in further experiments unless stated otherwise.

FTI treatment of H4 cells overexpressing αSyn resulted in increased levels of both the proform/single chain and the heavy chain of CTSD and CTSB (Additional file 1: Fig. S5c, d). Furthermore, treatment of αSyn-overexpressing H4 cells with FTI significantly increased the lysosomal activity of CTSD (measured in lysosome-enriched fractions of the cells), CTSL, and CTSB (assessed by live cell activity assays) (Fig. 3d). Furthermore, confocal microscopic analysis of cells co-stained with LAMP2 and CTSD or CTSB showed that lysosomal localisation of the cathepsins, indicated by colocalization with the lysosomal marker LAMP2 (Pearson correlation coefficient) and lysosomal intensity, was restored upon FTI treatment (Fig. 3e, f). These results support the hypothesis that αSyn interferes with hydrolase trafficking towards the lysosome. This effect could be compensated by treatment with FTI, restoring the cellular localization and enzymatic activity of CTSD, CTSB and CTSL.

### Rescue of lysosomal trafficking and activity of cathepsins in PD DA-iPSn (*SNCA* A53T and 3 × *SNCA*) by boosting intracellular protein transport

**Fig. 4:**
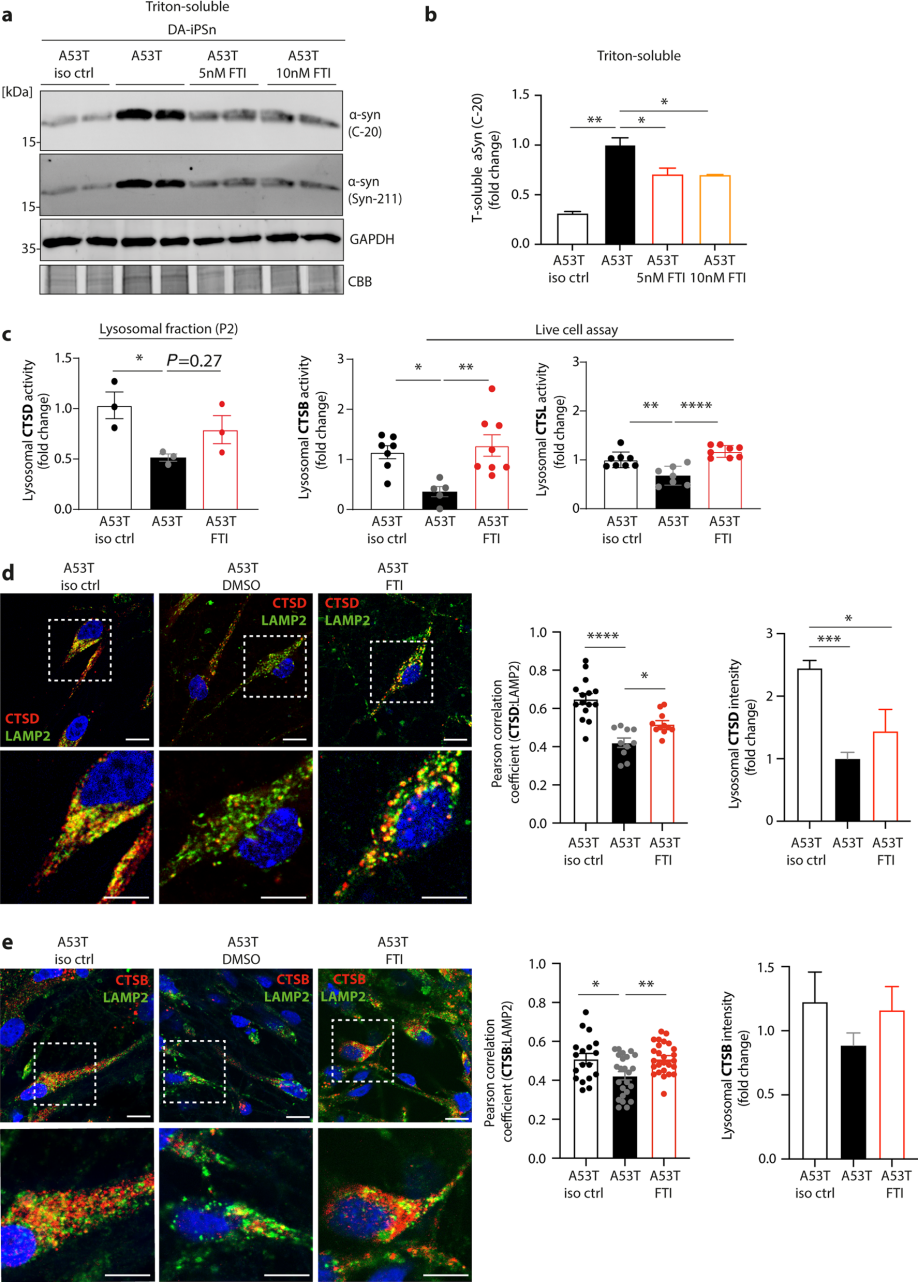
Improved trafficking and activity of cathepsins by FTI in DA-iPSn harbouring A53T mutation. **a** Western blot analysis of αSyn by utilizing C-20 and Syn-211 antibodies in DA-iPSn. A53T neurons were cultured with DMSO, or 5 or 10 nM FTI for 7 days with medium change every day. A53T isogenic control (iso ctrl) treated with DMSO was used as a positive control. **b** αSyn quantification of Western blots. Signal intensities of αSyn (detected with C-20 antibody) were normalized to the GAPDH signal and expressed as fold change, compared to A53T mutant neurons (*n* = 3). **c** CTSD activity was assessed in lysosome-enriched fractions of DA-iPSn with A53T mutation, FTI-treated A53T neurons and A53T iso ctrl (*n* = 3). Lysosomal activity of CTSB and CTSL was assessed in living A53T mutant neurons with or without  FTI treatment as well as A53T iso ctrl neurons (*n* = 4–8). **d** Left: Representative immunofluorescence images of A53T neurons cultured with DMSO or 5 nM FTI and respective iso ctrl. Neurons were stained for CTSD (red) and co-stained with LAMP2 (green). Nucleus is shown in blue. Scale bars: 10 µm. Right: Quantification of CTSD:LAMP2 co-staining by determining Pearson correlation coefficient, and lysosomal CTSD intensity analysis in A53T iso ctrl and A53T mutant neurons treated for 7 days with DMSO or FTI (Pearson correlation coefficient: *n* = 10–15 individual cells per group; lysosomal CTSD intensity analysis: *n* = 8–12 individual cells per group). **e** Left: Representative immunofluorescence images of A53T neurons cultured with DMSO or 5 nM FTI and iso ctrl. Neurons were stained for CTSB (red) and co-stained with LAMP2 (green). Nucleus is shown in blue. Scale bars: 10 µm. Right: Quantification of CTSB:LAMP2 co-staining by determining Pearson correlation coefficient, and lysosomal CTSB intensity analysis in A53T iso ctrl and A53T mutant neurons treated for 7 days with DMSO or FTI (Pearson correlation coefficient: *n* = 18–26 individual cells per group; lysosomal CTSB intensity analysis: *n* = 16–21 individual cells per group). Statistical analyses were performed by using one-way ANOVA together with a Tukey’s post-hoc test with ****P < 0.0001, ***P < 0.001, **P < 0.01, *P < 0.05

Next, we analysed the rescue effects of the LNK-754 (FTI) compound on lysosomal cathepsins in PD patient DA-iPSn. Improved lysosomal GCase activity and a decrease in αSyn levels after FTI application have been recently demonstrated in A53T DA neurons [36]. Western blotting demonstrated that FTI treatment at both concentrations of 5 and 10 nM resulted in decreased αSyn protein levels in A53T DA-iPSn (Fig. 4a, b). As shown in Fig. 2f and i, A53T neurons displayed lower levels of mature forms of CTSD, CTSL, and CTSB compared to the isogenic control. This is in line with the decline in proteolytic activity of CTSD (measured in lysosome-enriched fractions) as well as CTSL and CTSB (assessed via live cell activity assays) for neurons harbouring αSyn A53T mutation (Fig. 4c). Remarkably, application of 5 nM FTI for 7 days restored the activity of all three lysosomal enzymes (Fig. 4c). Immunofluorescence staining revealed increased colocalization of CTSD and CTSB with LAMP2 as well as lysosomal localisation (signal intensity) within neurons corrected for the A53T mutation (isogenic control) in comparison to mutant A53T DA-iPSn (Fig. 4d, e). Interestingly, the application of FTI in mutant A53T neurons increased the overall lysosomal trafficking of CTSD and CTSB, indicated by the significant elevation of colocalisation with the lysosomal marker LAMP2 analysed by Pearson correlation coefficient as well as by a trend of increase of lysosomal signal intensity (Fig. 4d, e).

Similar to the observations in the DA-iPSn A53T mutant cell model, FTI treatment (5 nM, 7 days) of 3 × *SNCA* cells significantly decreased the otherwise elevated protein levels of soluble and insoluble αSyn detected with the non-pathology-related Syn1 and pathology-related LB509 antibodies (Additional file 1: Fig. S6a-c). Immunofluorescence analysis of the lysosomal marker LAMP2 and CTSD showed decreased tendency of LAMP2:CTSD colocalization and diminished lysosomal CTSD intensity (Additional file 1: Fig. S6d). In the case of CTSB, both LAMP2:CTSB colocalization and CTSB lysosomal intensity were significantly decreased in the mutant cells compared to the isogenic control (Additional file 1: Fig. S6e). Treatment of 3 × *SNCA* cells with 5 nM FTI restored lysosomal trafficking of CTSB, indicated by increased CTSB:LAMP2 colocalisation and enhanced lysosomal CTSB signal intensity (Additional file 1: Fig. S6e).

Overall, these data indicate that the trafficking and maturation defects of CTSD, CTSB, and CTSL in midbrain neurons harbouring synucleinopathies (A53T *SNCA* mutation and *SNCA* gene triplication), could be rescued by enhancing hydrolase transport towards the lysosomes via activation of the ykt6 pathway, using the small compound FTI.

### Restoring impaired trafficking of CTSD, CTSB, and CTSL in mice harbouring *SNCA* A53T point mutation in DA neurons

**Fig. 5:**
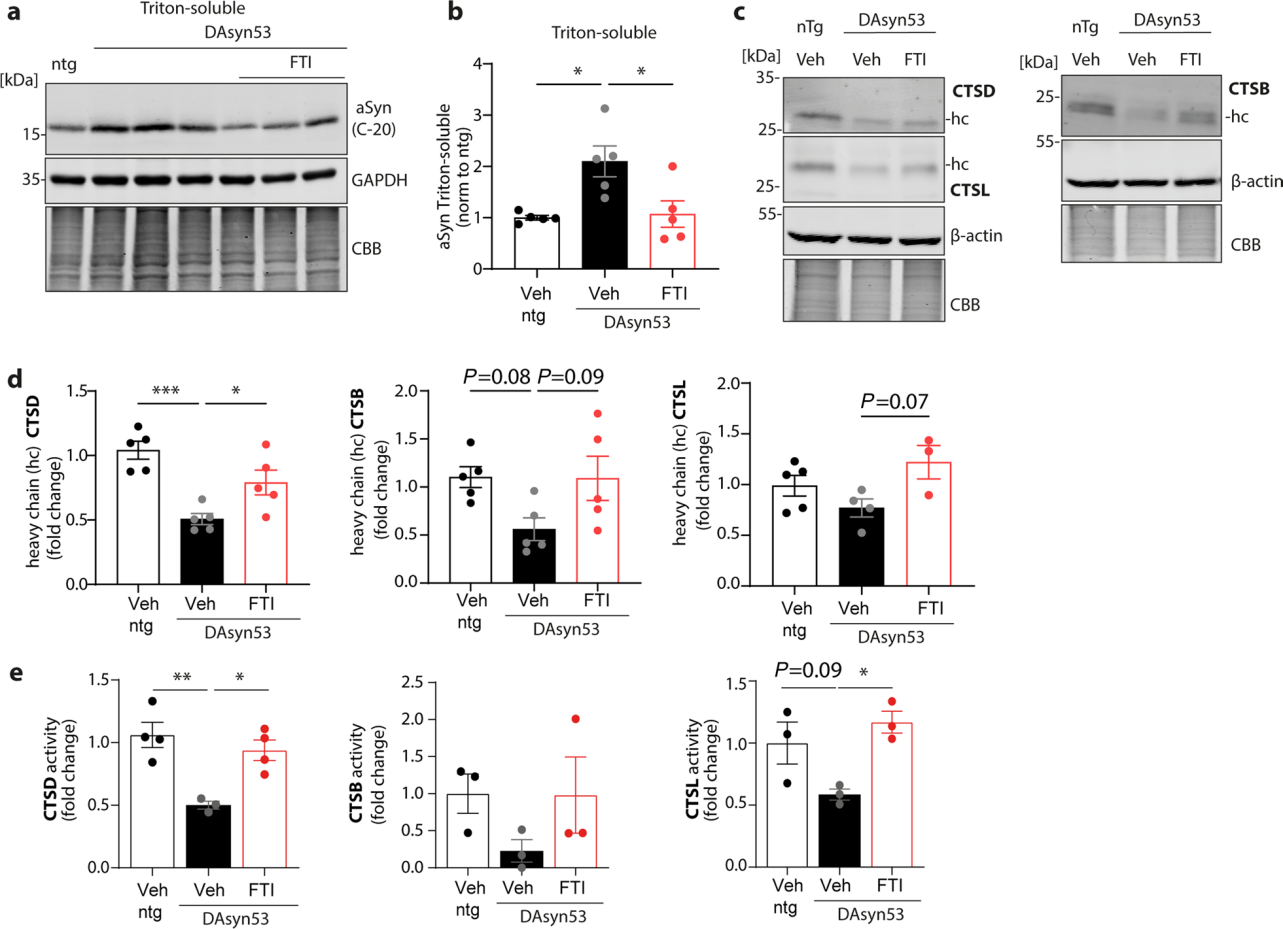
Rescue of disrupted cathepsin maturation and activity in mice overexpressing αSyn A53T in dopaminergic neurons. **a** Western blot analysis of αSyn detected with C-20 antibody in thalamus/midbrain Triton-soluble samples of non-transgenic (ntg) mice and mice overexpressing human A53T in dopaminergic neurons (DA_syn53_). DA_syn53_ mice were treated with FTI for 26 days. **b** Quantification of αSyn signal intensities of ntg, DA_SYN53_ and FTI-applied DA_SYN53_ mice. Each αSyn signal (detected with C-20 antibody) was normalized to the corresponding GAPDH signal and displayed as fold change, compared to vehicle ntg mice (*n* = 5). **c** Representative Western blot analyses of thalamus/midbrain samples of ntg, DA_syn53_ and DA_syn53_ mice treated with FTI stained for CTSD, CTSL as well as CTSB. **d** Corresponding quantifications of Western blot analyses of mature forms (heavy chain, hc) of CTSD (left), CTSB (middle) and CTSL (right). Signal intensities were normalized to β-actin signals and expressed as fold change, compared to ntg (*n* = 3–5). **e** Enzymatic activity assay for CTSD, CTSB, and CTSL in whole lysates from thalamus/midbrain mouse brain samples (*n* = 3–4). Statistical analyses were performed by using one-way ANOVA together with Tukey’s multiple comparison test. ***P < 0.001, **P < 0.01, *P < 0.05

Based on our findings in different cell culture models, we tested whether FTI is capable of restoring correct lysosomal trafficking of CTSD, CTSB and CTSL in vivo. For this, we used an established mouse model that expresses human A53T *SNCA* within DA neurons (DA_SYN53_) [46]. These mice show increased αSyn levels compared to non-transgenic (ntg) littermates (Fig. 5a, b; Additional file 1: Fig. S7a,b). DA_SYN53_ mice were i.p. injected daily with FTI for 26 days. Midbrain/thalamic region extracts of FTI-treated DA_SYN53_ mice showed reduced αSyn levels when compared to samples of DA_SYN53_ mice without FTI treatment. The reduced αSyn protein levels found in FTI-treated DA_SYN53_ animals were comparable to the αSyn protein levels seen in ntg animals (Fig. 5a, b; Additional file 1: Fig. S7a, b). Western blot analysis of cathepsins indicated a reduction of mature forms of all three cathepsins in DA_SYN53_ mice; however, only CTSD level yielded significant differences (Fig. 5c, d). FTI treatment restored the maturation of CTSD to levels found in ntg mice. Similarly, for CTSB and CTSL, an increase of mature forms was observed in FTI-treated DA_SYN53_ animals; however, neither of those reached a significant level (Fig. 5c, d). To determine the functional effects of FTI on the lysosomal system, we measured activities of cathepsins in midbrain/thalamic regions of DA_SYN53_ mice and found that the CTSD activity was significantly diminished in DA_SYN53_ mice and that FTI treatment restored its activity almost to the level observed in ntg mice (Fig. 5e, left). Analyses for CTSB and CTSL activity in brain tissue revealed similar results (Fig. 5e, middle and right). Overall, these data underline the potential of FTI treatment in vivo to lower αSyn level and rescue lysosomal cathepsin deficiencies by restoring the enzymatic functions of all here analysed lysosomal cathepsins CTSD, CTSB, and CTSL.

## Discussion

Multiplications or mutations (e.g. A53T) within the *SNCA* gene have been shown to result in massive αSyn aggregation and accumulation that impact PD severity [3, 4, 6]. The ALP is thereby considered one of the main degradation pathways for αSyn, next to the ubiquitin–proteasome system [12, 47, 48]. Dysfunction of the ALP results in aberrant aggregation of several proteins including the small synaptic protein αSyn, triggering PD pathology [49, 50]. Cathepsins have been discovered to be the key players in αSyn metabolism and directly linked to its clearance in vitro and in vivo [24, 26]. In line with this, we show that the inhibition of the enzymatic activity of the most abundant lysosomal hydrolases CTSD, CTSB, and CTSL [22], by chemical compounds, enhances the formation of soluble αSyn in H4 cells and PD patient-derived DA neurons harbouring αSyn triplication (Additional file 1: Fig. S8a–d) as well as insoluble αSyn forms in PD iPS-DA neurons (3 × *SNCA*) (Additional file 1: Fig. S8e, f). Interestingly, the inhibition of cathepsins in DA-iPSn corrected for the αSyn triplication (isogenic control) did not affect αSyn protein levels (Additional file 1: Fig. S8g–j). Clearance of αSyn is not only limited by the ALP system but also regulated by the proteasome favouring the elimination of soluble, short-lived αSyn forms [47, 51, 52]. In contrast, autophagy is considered to be responsible for the bulk degradation of longer-lived macromolecules and thus aggregated αSyn [11, 47, 53–55]. Since the isogenic control cells do not display a significant amount of αSyn protein (Fig. 2a, b; Additional file 1: Figs. S4a, b and S8g–j), the αSyn turnover might be controlled mainly by the proteasomal system.

Our results show that PD patient-derived dopaminergic neurons exhibiting αSyn aggregation display impaired lysosomal trafficking of cathepsins, resulting in reduced proteolytic activity of cathepsins in the lysosome (Figs. 2 and 4). Earlier studies already identified reduced activity of lysosomal enzymes in PD patients and various PD models [56, 57]. For instance, the activity of GCase, an important lysosomal enzyme that hydrolyses glucosylceramide into glucose and ceramide [58], was found to be reduced in brain tissues of PD patients, including the SN [59, 60]. The functional loss of GCase was shown to promote the formation of αSyn accumulation with neurotoxic properties and aggregated αSyn was proposed to reduce GCase activity through the disruption of hydrolase trafficking towards the lysosome in iPSC-derived midbrain neurons. In the same study, there was a decrease in the lysosomal CTSB activity within PD neurons [32]. In line with this, our findings show reduced enzymatic activity for CTSB as well as for CTSD and CTSL in H4 cells overexpressing αSyn and in DA-iPSn with αSyn triplication (3 × *SNCA*) and A53T point mutation (Figs. 1f, 2e and 4c). Reduced activity of CTSD has also been found in whole-brain PD samples [57] and in the frontal cortex of PD and DLB patients without changes in mRNA expression [60]. Supporting these findings, our data indicate trafficking defects of lysosomal cathepsins instead of changes on a transcriptional level (Additional file 1: Figs. S1a and S4h, i). Furthermore, reductions of both the activity and the protein level of CTSD have been demonstrated in biological fluids such as cerebrospinal fluid (CSF) [61] and plasma [62]. The reduced CTSD maturation and activation could be due to decreased activity of GCase leading to reduced ceramide levels, which are considered to influence CTSD maturation [63]. Moreover, low levels of CTSB have been found in PD CSF samples [64], indicating that reduced activities of CTSD and CTSB are strongly associated with PD and other synucleinopathies. Interestingly, genome-wide association studies discovered that CTSD and CTSB are also susceptible genes for developing PD [16, 21].

We demonstrate here that next to CTSD and CTSB, lysosomal CTSL activity is strongly reduced in PD-derived iPSn (Figs. 2e and 4c). Although CTSL is considered to be the most efficient cathepsin in the degradation of fibrillary αSyn structures [26, 65], studies are yet to demonstrate its altered enzymatic function in PD patient-derived cells. To date, 11 cysteine cathepsins are known, which share similar cleavage properties, thus being capable of compensating for the loss-of-function of other individual cathepsins [66]. Most studies analyzed the activity of cathepsins in whole cells or tissues, which did not accurately reflect the activity within the acidic lysosomal compartment given that cathepsins trapped within the secretory pathway can be activated post-lysis in acidic buffers optimized for activity assays.

Our data show that the lysosomal activities of CTSD, CTSB, and CTSL are reduced in iPSC-derived DA neurons of PD patients. Moreover, the lack of activity of all three of the main lysosomal hydrolases, which are directly involved in αSyn degradation, results in a failure of efficient lysosomal degradation and accelerates αSyn accumulation. In a reciprocal manner, this has a negative impact on the cellular trafficking and maturation of cathepsins along the secretory pathway, as well as other hydrolases, creating a vicious cycle of ineffective αSyn clearance.

Importantly, lysosomal cathepsins do not only degrade αSyn, but also other substrates, including numerous aggregation-prone proteins associated with numerous neurodegenerative diseases: (i) amyloid-beta precursor protein (APP) and (ii) microtubule-associated protein tau that are both related to Alzheimer’s disease [67–69], (iii) huntingtin related to Huntington’s disease [70, 71], and (iv) prion protein related to the prion protein diseases [72, 73]. Thus, lack of activity of cathepsins could also induce the aggregation of pathology-associated substrates other than αSyn and contribute to neurodegeneration.

Here we show that treatment with FTI rescued the impaired lysosomal maturation and activity in H4 cells (Fig. 3d-f), in PD-associated midbrain neurons (Fig. 4c–e) and in mouse brain samples overexpressing pathological αSyn (Fig. 5e). FTI has been demonstrated to enhance a lysosomal stress response pathway by activating the small SNARE protein ykt6, boosting lysosomal enzyme trafficking and lysosomal function [36, 37]. Enhancing the transport of hydrolases towards the lysosome by FTI decreased αSyn protein levels and pathology-associated αSyn conformers (insoluble and positive for LB509 αSyn antibody) in our cell models (Figs. 3b, c; 4a, b; Additional file 1: Fig. S6a, b) and in vivo (Fig. 5a, b; Additional file 1: Fig. S7a, b), which is in line with a previous study [36]. Tackling hydrolase trafficking has been shown to be a promising therapeutic strategy to rescue aberrant αSyn pathology [32, 41, 74]. This notion is further supported by the findings that overexpression of a key mediator of vesicular trafficking Rab1a was able to restore lysosomal trafficking and activity, consequently leading to decreased αSyn pathology in patient-derived neurons [32]. Furthermore, a recent study demonstrated that treatment with a recombinant proCTSD reduces not only pathological αSyn in midbrain DA-iPSn, but also pathological species found in lysosomes derived from PD patient iPSn. Further, treatment of iPSC-derived neurons harboring A53T *SNCA* mutation with proCTSD restores and improves endo-lysosomal function, which is distressed by accumulated αSyn [25].

## Conclusion

In PD-derived midbrain neurons as well as in an in vivo model harbouring synuclein pathology, the transport of cathepsins towards the lysosome is disturbed in an αSyn-dependent manner. Consequently, this resulted in diminished lysosomal proteolytic activity of the αSyn-degrading enzymes CTSD, CTSB, and CTSL, which could be a further driver of αSyn pathology. We here suggest that improving maturation and lysosomal function of cathepsins by boosting their lysosomal transport in an ykt6-dependent manner, might have a therapeutic potential to lower αSyn level in PD and other synucleinopathies (see graphical abstract).

## Supplementary Information

**Additional file 1**. **Fig. S1** H4 neuroglioma cells overexpressing αSyn under the tetracycline responsive promoter. **Fig. S2** Representative pictures of pluripotency marker staining of induced pluripotent stem cells (iPSCs). **Fig. S3** Characterization of induced pluripotent stem cell-derived dopaminergic neurons (DA-iPSn). **Fig. S4** Triplication and mutation within the *SNCA* gene cause αSyn accumulation and decreased cathepsin maturation. **Fig. S5** Effect of FTI treatment on αSyn and cathepsin levels in H4 cells.** Fig. S6 **Improved cathepsin trafficking by FTI in 3×*SNCA* DA-iPSn. **Fig. S7 **Farnesyltransferase inhibitor (FTI) treatment decreases the level of soluble αSyn in mice overexpressing αSyn A53T in dopaminergic neurons. **Fig. S8** Inhibition of lysosomal proteases CTSD, CTSL and CTB causes αSyn accumulation.

## Data Availability

All data supporting the conclusions of this article are included within the article and in additional files provided.

## Abbreviations

3 × *SNCA*: αSyn triplication
ALP: Autophagy-lysosomal pathway
BafA1: Bafilomycin A1
TUBB3: Beta-III tubulin
CSF: Cerebrospinal fluid
Ctrl: Control
CTSB: Cathepsin B
CTSD: Cathepsin D
CTSL: Cathepsin L
DA: Midbrain dopaminergic neurons
DOX: Doxycycline
ER: Endoplasmic reticulum
FACS: Flow cytometry analysis
FCS: Fetal calf serum
FTI: Farnesyltransferase inhibitors
GCase: Beta-glucocerebrosidase
Iso ctrl: Isogenic control
LSD: Lysosomal storage disorder
PIC: Protease inhibitor cocktail
KO: Knockout
NCL-10: Neuronal ceroid lipofuscinoses type 10
Ntg: Non-transgenic
PepA: Pepstatin A
PD: Parkinson’s disease
iPSC: Induced pluripotent stem cell
iPSn: Induced pluripotent stem cell-derived neuron
LAMP2: Lysosome-associated membrane protein 2
SN: Substantia nigra
SNARE: N-ethylmaleimide-sensitive-factor attachment protein receptor
Tet: Tetracycline
TH: Tyrosine hydroxylase
