# Supplementary material for: Reciprocal effects of alpha-synuclein aggregation and lysosomal homeostasis in synucleinopathy models
Source: Transl Neurodegener. 2023 Jun 13;12:31. doi: 10.1186/s40035-023-00363-z (PMC10262594; doi:10.1186/s40035-023-00363-z)
Supplement: Supplementary file 1 — Additional file 1. Fig. S1 H4 neuroglioma cells overexpressing αSyn under the tetracycline responsive promoter. Fig. S2 Representative pictures of pluripotency marker staining of induced pluripotent stem cells (iPSCs). Fig. S3 Characterization of induced pluripotent stem cell-derived dopaminergic neurons (DA-iPSn). Fig. S4 Triplication and mutation within the SNCA gene cause αSyn accumulation and decreased cathepsin maturation. Fig. S5 Effect of FTI treatment on αSyn and cathepsin levels in H4 cells. Fig. S6 Improved cathepsin trafficking by FTI in 3×SNCA DA-iPSn. Fig. S7 Farnesyltransferase inhibitor (FTI) treatment decreases the level of soluble αSyn in mice overexpressing αSyn A53T in dopaminergic neurons. Fig. S8 Inhibition of lysosomal proteases CTSD, CTSL and CTB causes αSyn accumulation. [file 40035_2023_363_MOESM1_ESM.docx]

**
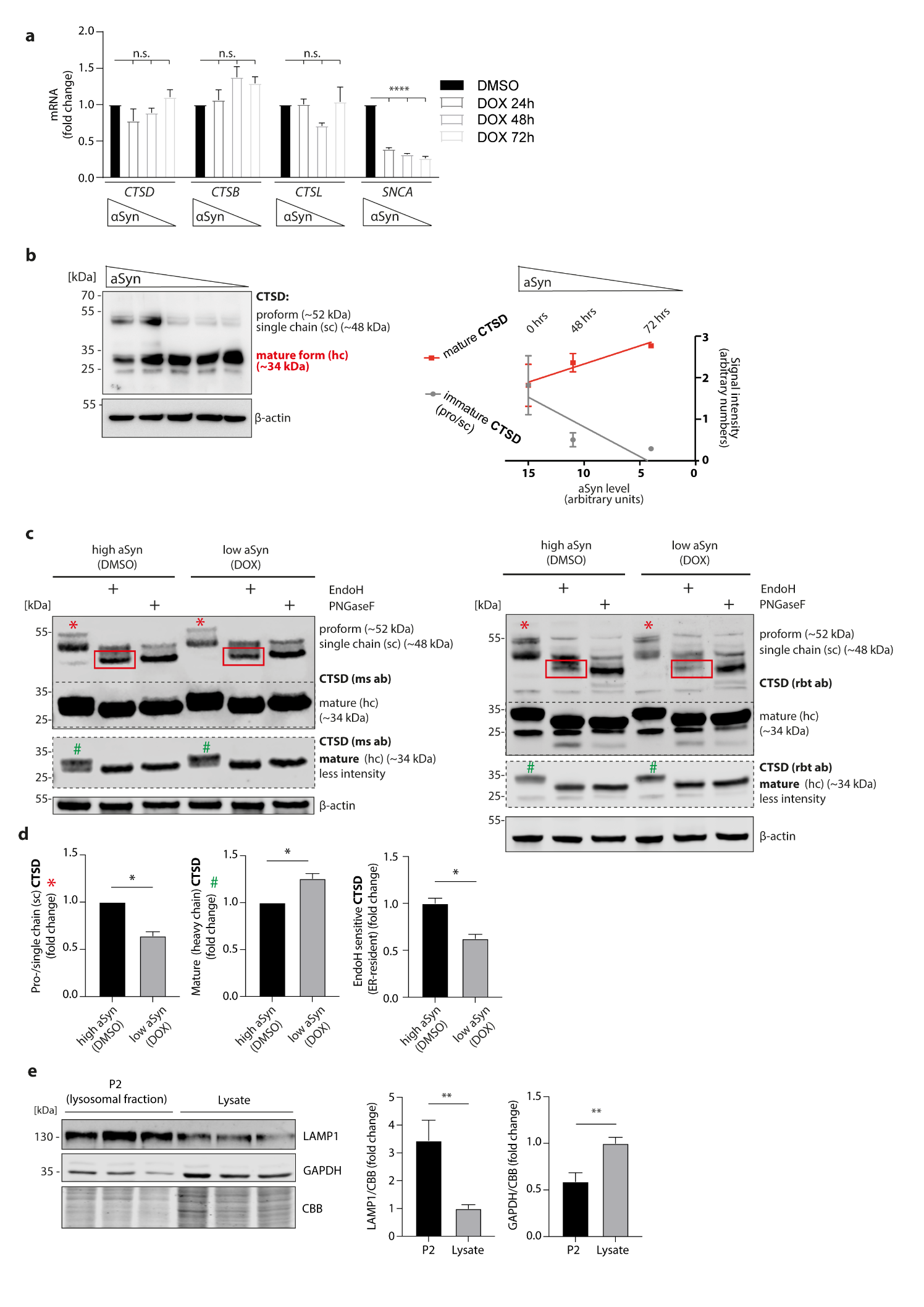
Fig. S1** H4 neuroglioma cells overexpressing αSyn under the tetracycline responsive promoter.

**a** mRNA expression levels of cathepsins (CTSD, CTSB, and CTSL), and αSyn in H4 cells determined by RT-qPCR. αSyn expression was down-regulated by 24 h, 48 h, and 72 h DOX treatment. Signals were normalized to the mean of GAPDH and β-actin and expressed as fold change, compared to samples treated with DMSO for 72h (n=3).

**b** Representative western blot analysis illustrating the divergence of the levels of pro-form/single chain (sc) and mature (heavy chain, hc) form of CTSD in αSyn overexpressing H4 cells. Down-regulation of αSyn expression by treatment of the cells with DOX for 48 or 72 h resulted in a gradual decrease of the immature (pro-form/sc), and increase of the mature form (heavy chain, hc) of the enzyme.

**c** Western blot analysis of pro-form/single chain (sc) and mature (heavy chain, hc) form of CTSD in H4 cells high in αSyn (DMSO) and low αSyn (DOX). Prior to loading, EndoH treatment was performed of the indicated samples. PNGaseF treatment was included as control for complete digest of carbohydrates. For the detection of CTSD two antibodies derived from different host species (mouse (ms) and rabbit (rbt)) were implemented. β-actin staining was used as loading control. The bands analysed are marked with *, #, or are framed.

**d** Quantification of western blots shown in Fig.c. Significant decrease in the pro-form/single chain of CTSD is detected in DOX treated cells (low αSyn) compared to samples of αSyn overexpressing cells (DMSO treated) (left; bands analysed are marked with * on the blots shown above). On the contrary, the level of mature/heavy chain (hc) of the enzyme was significantly increased in cells showing low αSyn expression (DOX treated) (middle; bands analysed are marked with # on the blots shown above). The levels of EndoH sensitive (endoplasmatic reticulum (ER) resident) form of the enzyme shows decreased levels in cells with low αSyn levels (DOX treatment) (right; bands analysed are marked with a frame on the blots shown above).

**e** Quality control of lysosomal enrichment of H4 cells. Western blot analysis shows increase in the levels of the lysosomal marker LAMP1 and decrease of GAPDH in the lysosome-enriched P2 fractions compared to whole cell lysates (*n*=3).

Statistical analyses were performed by using two-tailed unpaired Student’s t-tests (d and e) or one-way ANOVA together with Dunnett’s multiple comparison test toward DMSO control (a). **** *P* < 0.0001, ** *P* < 0.01, * *P* < 0.05

**
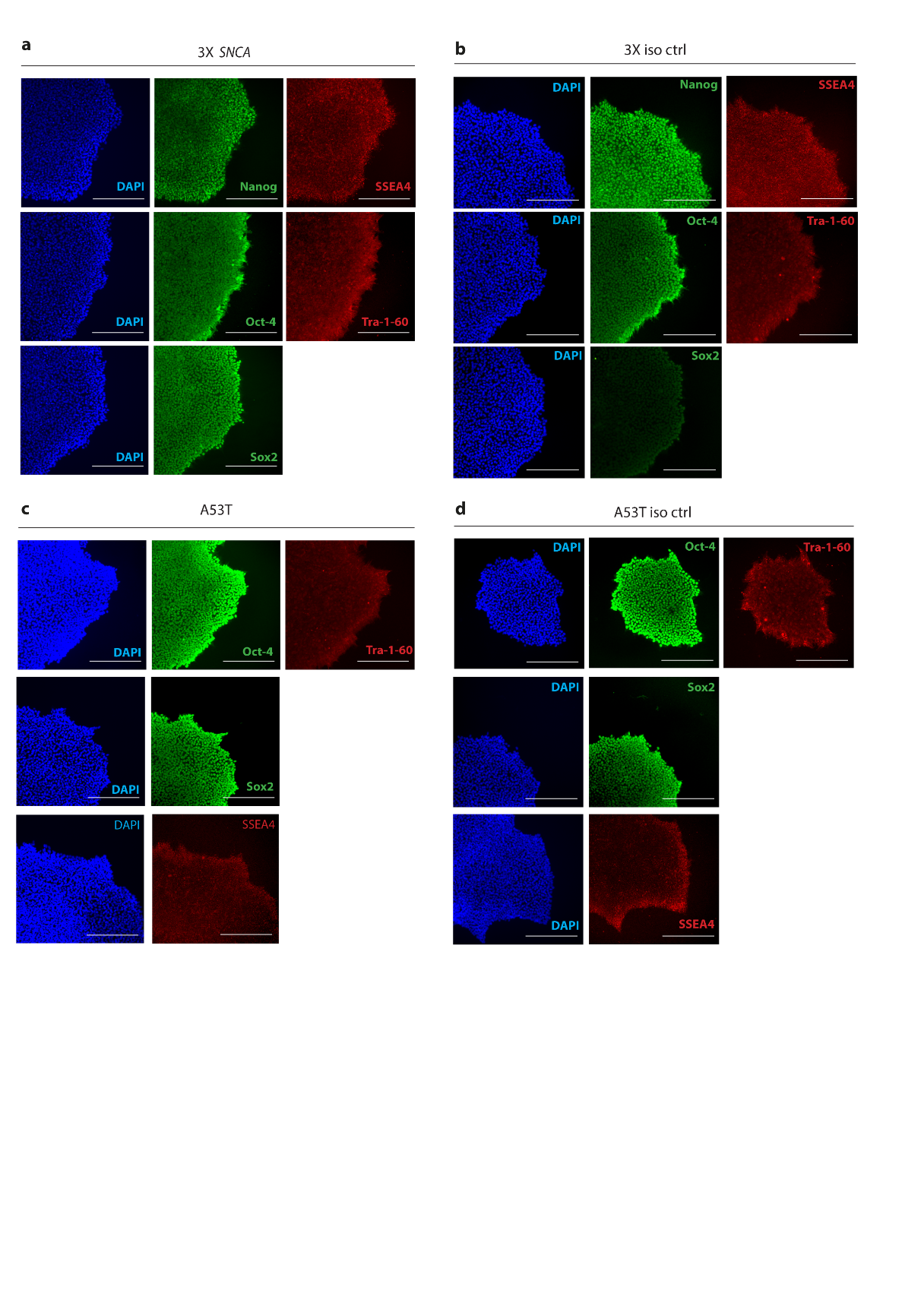
**

**Fig. S2:** Representative pictures of pluripotency marker staining of induced pluripotent stem cells (iPSCs).

Pluripotency of iPSCs was confirmed by immunofluorescence staining of the stem cell markers Nanog, Oct4, Tra-1-60, SSEA4, and SOX2 in 3×*SNCA* and A53T mutant cells **(a,c)** and their respective iso ctrl lines **(b, d)** (scale bar=300µm).

**
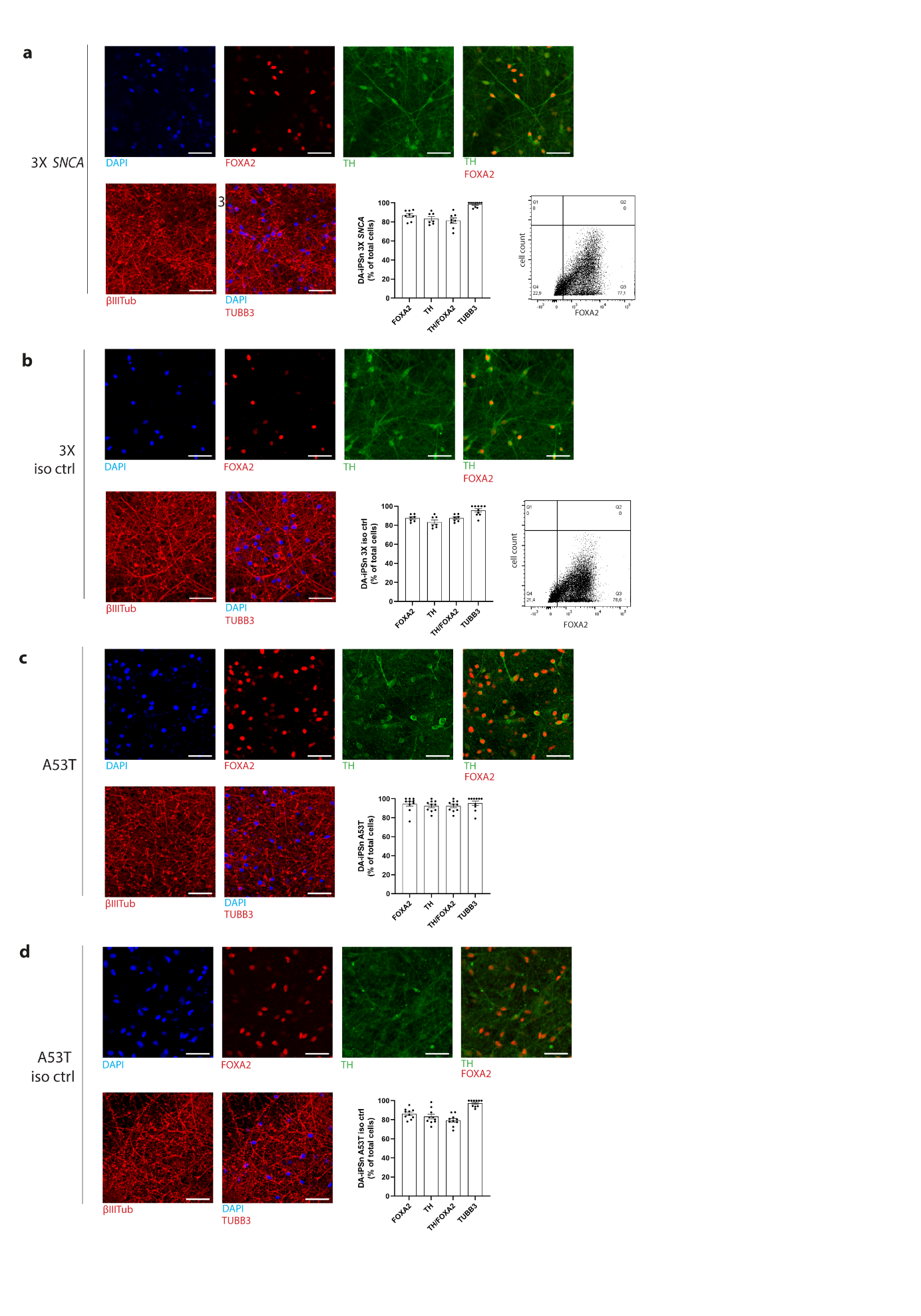
**

**Fig. S3** Characterization of induced pluripotent stem cell-derived dopaminergic neurons (DA-iPSn).

**a** Representative immunofluorescence images of 3×*SNCA* neurons stained for midbrain specific and neuronal markers. The midbrain specific FOXA2 (upper row, red) and dopaminergic neuron-specific tyrosine hydroxylase (upper row, TH, green) was expressed by the majority of the cells. Similarly, almost 100 percent of the cells were positive for the neuronal specific beta-III tubulin (second row, red, TUBB3). The percentage of cells expressing the analysed markers is shown in the graph (each data point of the scatter plots represents a field of view, data is shown as mean ± SEM) (scale bar=50µm). 77.1 percent of cells analysed by flow cytometry were positive for FOXA2.

**b** Representative immunofluorescence images of 3X iso ctrl neurons stained for midbrain specific and neuronal markers. The midbrain specific FOXA2 (upper row, red) and dopaminergic neuron-specific tyrosine hydroxylase (upper row, TH, green) was expressed by the majority of the cells. Similarly, almost 100 percent of the cells were positive for the neuronal specific beta-III tubulin (second row, red, TUBB3). The percentage of cells expressing the analysed markers is shown in the graph (each data point of the scatter plots represents a field of view, data is shown as mean ± SEM) (scale bar=50µm). 78.6 percent of cells analysed by flow cytometry were positive for FOXA2.

**c** Representative immunofluorescence images of A53T mutant neurons stained for midbrain specific and neuronal markers. The midbrain specific FOXA2 (upper row, red) and dopaminergic neuron-specific tyrosine hydroxylase (upper row, TH, green) was expressed by more, than 90 percent of the cells. Similarly, almost 100 percent of the cells were positive for the neuronal specific beta-III tubulin (second row, red, TUBB3). The percentage of cells expressing the analysed markers is shown in the graph (each data point of the scatter plots represents a field of view, data is shown as mean ± SEM) (scale bar=50µm).

**d** Representative immunofluorescence images of A53T iso ctrl neurons stained for midbrain specific and neuronal markers. The midbrain specific FOXA2 (upper row, red) and dopaminergic neuron-specific tyrosine hydroxylase (upper row, TH, green) was expressed by the majority of the cells. Similarly, almost 100 percent of the cells were positive for the neuronal specific beta-III tubulin (second row, red, TUBB3). The percentage of cells expressing the analysed markers is shown in the graph (each data point of the scatter plots represents a field of view, data is shown as mean ± SEM) (scale bar=50µm).

**
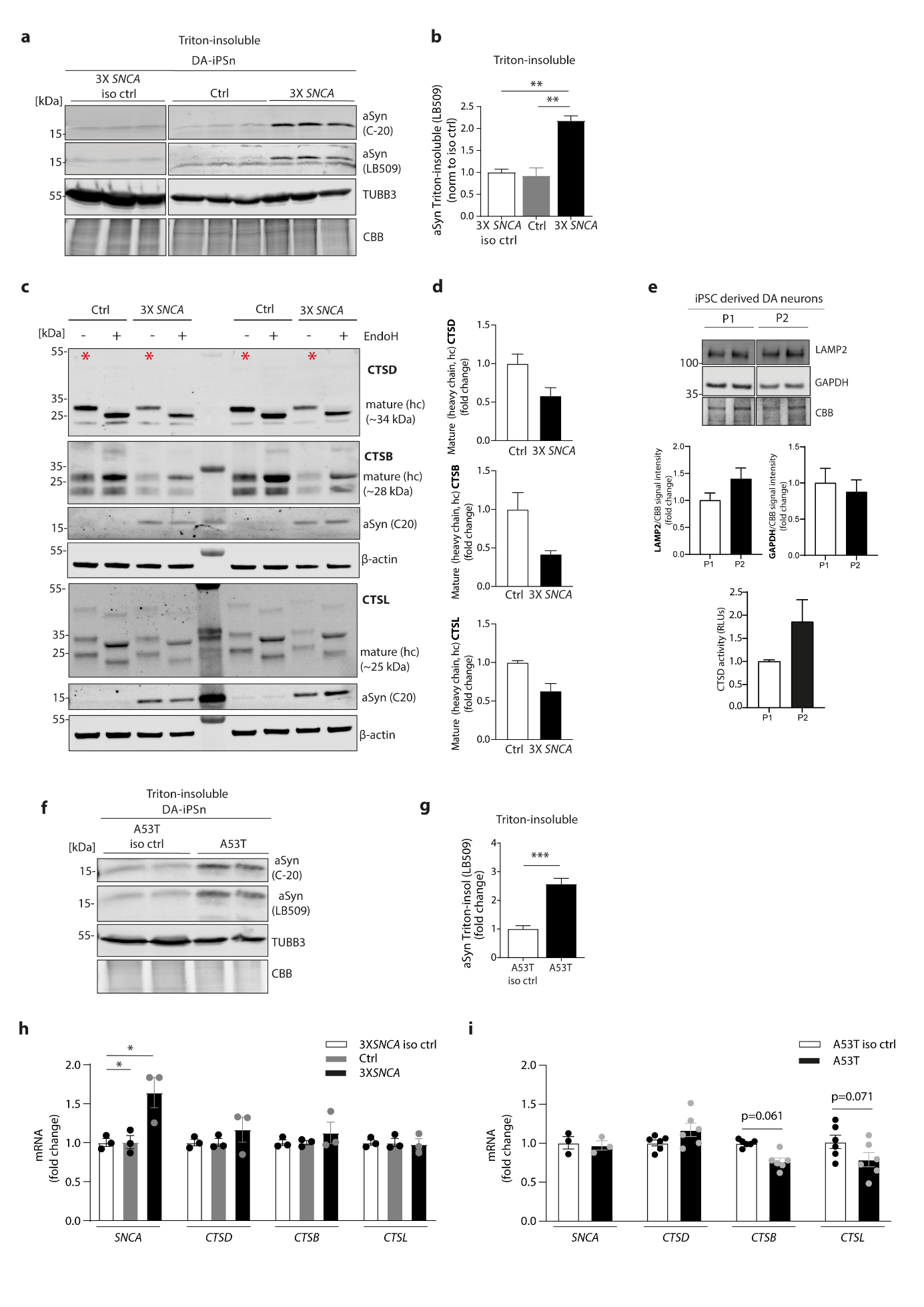
**

**Fig. S4** Triplication and mutation within the *SNCA* gene cause αSyn accumulation and decreased cathepsin maturation.

**a** Immunoblot analyses of Triton-insoluble fraction of neurons for 3×*SNCA* iso ctrl*,* ctrl and 3×*SNCA* mutation. Signals for αSyn were detected by C-20 and LB509 primary antibodies. TUBB3 and CBB were used to show equal protein load.

**b** Respective quantification of western blot analysis in Triton-insoluble fraction of DA neurons carrying 3×*SNCA*, and respective iso ctrl and ctrl. αSyn signals were detected with the use of the LB509 αSyn antibody. Values were normalized to CBB and compared to 3×*SNCA* iso ctrl (*n*=3).

**c** Western blot analyses of αSyn, CTSD, CTSB, and CTSL in Triton-soluble fractions of 3×*SNCA* and ctrl DA-iPSn with and without EndoH digestion. β-actin served as loading control.

**d** Quantification of western blot analysis (shown in Fig.S4c) of heavy chain of CTSD, CTSB, and CTSL in non-EndoH treated (- EndoH; lanes marked with *) Triton-soluble fraction samples of 3×*SNCA* and ctrl DA neurons. Signals were normalized to β-actin and compared to ctrl.

**e** Quality control of lysosomal enrichment of DA-iPSn. Western blot analysis shows decreased level of GAPDH and increased level of the lysosomal marker LAMP2 in the lysosome-enriched P2 fractions compared to P1 fractions, containing unbroken cells, nuclei, and heavy mitochondria (graph on the left and middle, respectively. Signals were normalized to CBB and expressed as fold change compared to P1 fraction, *n*=2). CTSD activity assay shows increased activity of the enzyme in the lysosomes-enriched P2 fraction compared to P1 fraction (graph on right, data shown as normalized to P1, *n*=2).

**f** Western blot analyses of Triton-insoluble fraction of DA-iPSn A53T mutation and iso ctrl. Two different αSyn antibodies (C-20 and LB509) were used to detect aggregated αSyn levels. TUBB3 and CBB served as loading controls.

**g** Quantification of western blot analysis in Triton-insoluble fraction of DA neurons harbouring a A53T mutation and isogenic control. LB509 primary antibody was used to detect αSyn. Signals were normalized to TUBB3 and compared to A53T iso ctrl (*n*=3).

**h** mRNA levels of CTSD, CTSB and CTSL were determined by RT-qPCR in DA neurons of 3×*SNCA*, iso ctrl and healthy ctrl. Values were normalized to the mean of GAPDH and β-actin, and expressed as fold change, compared to the iso ctrl (*n*=3).

**i** mRNA levels in A53T neurons determined by RT-qPCR. Expression levels were normalized to GAPDH and β-actin and shown as fold change relative to the iso ctrl (*n*=3-6).

Statistical analyses were performed by using two-tailed unpaired Student’s t-tests (d-e, g and i) and one-way ANOVA together with Dunnett’s multiple comparison test towards iso ctrl (b and h). **** *P* < 0.0001, ** *P* < 0.01, * *P* < 0.05.

**
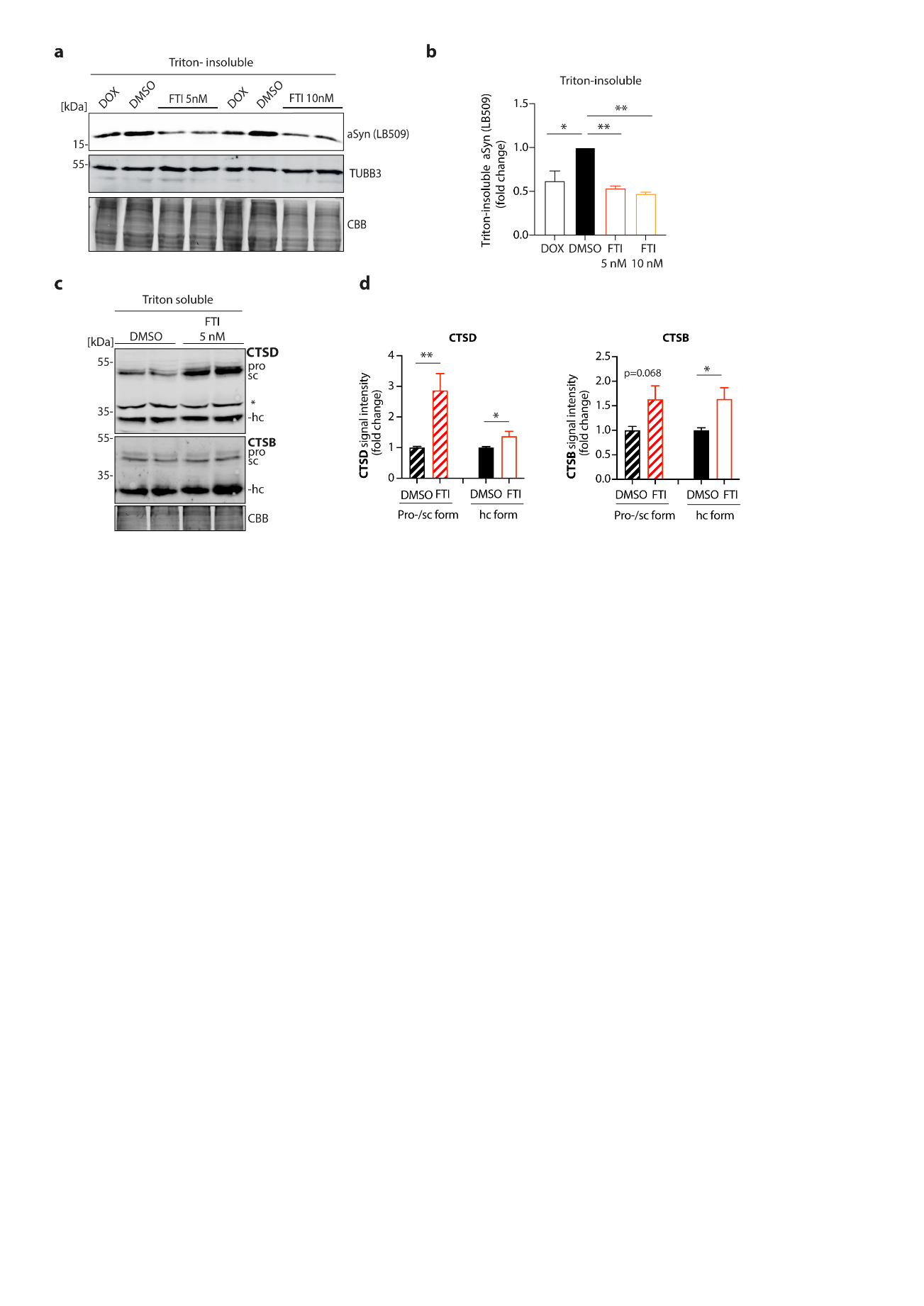
**

**Fig. S5** Effect of FTI treatment on αSyn and cathepsin levels in H4 cells.

**a** Western blot analysis of Triton-insoluble fractions of H4 cells low in αSyn (DOX treated), or high in αSyn (DMSO treated), with or without FTI treatment (5 or 10 nM). For detection of insoluble αSyn, the pathology-related LB509 antibody was used. TUBB3 served as loading control.

**b** Quantification of Triton-insoluble αSyn signals in H4 cells low in αSyn (DOX treated), or high in αSyn (DMSO treated), with or without FTI treatment (5 or 10 nM). Signals were normalized to TUBB3 and expressed as fold change, compared to DMSO treated (αSyn overexpressing) cells (*n*=4).

**c** Western blot analysis of the pro-form/single chain (sc) and heavy chain (hc) in Triton-soluble fractions of αSyn overexpressing H4 cells (DMSO treated) with or without 5 nM FTI treatment. CBB served as loading control.

**d** Quantification of CTSD (left) and CTSB (right) pro-form/single chain (sc) and heavy chain (hc) in Triton-soluble fractions of αSyn overexpressing H4 cells (DMSO treated) with or without 5 nM FTI treatment. Signals were normalized to CBB and expressed as fold change, compared to DMSO treated (αSyn overexpressing) cells (*n*=2-4).

Statistical analyses were performed by using one-way ANOVA together with Dunnett’s multiple comparison test towards DMSO treated samples (b) and two-tailed unpaired Student’s t-tests (d). ** *P* < 0.01, * *P* < 0.05.

**
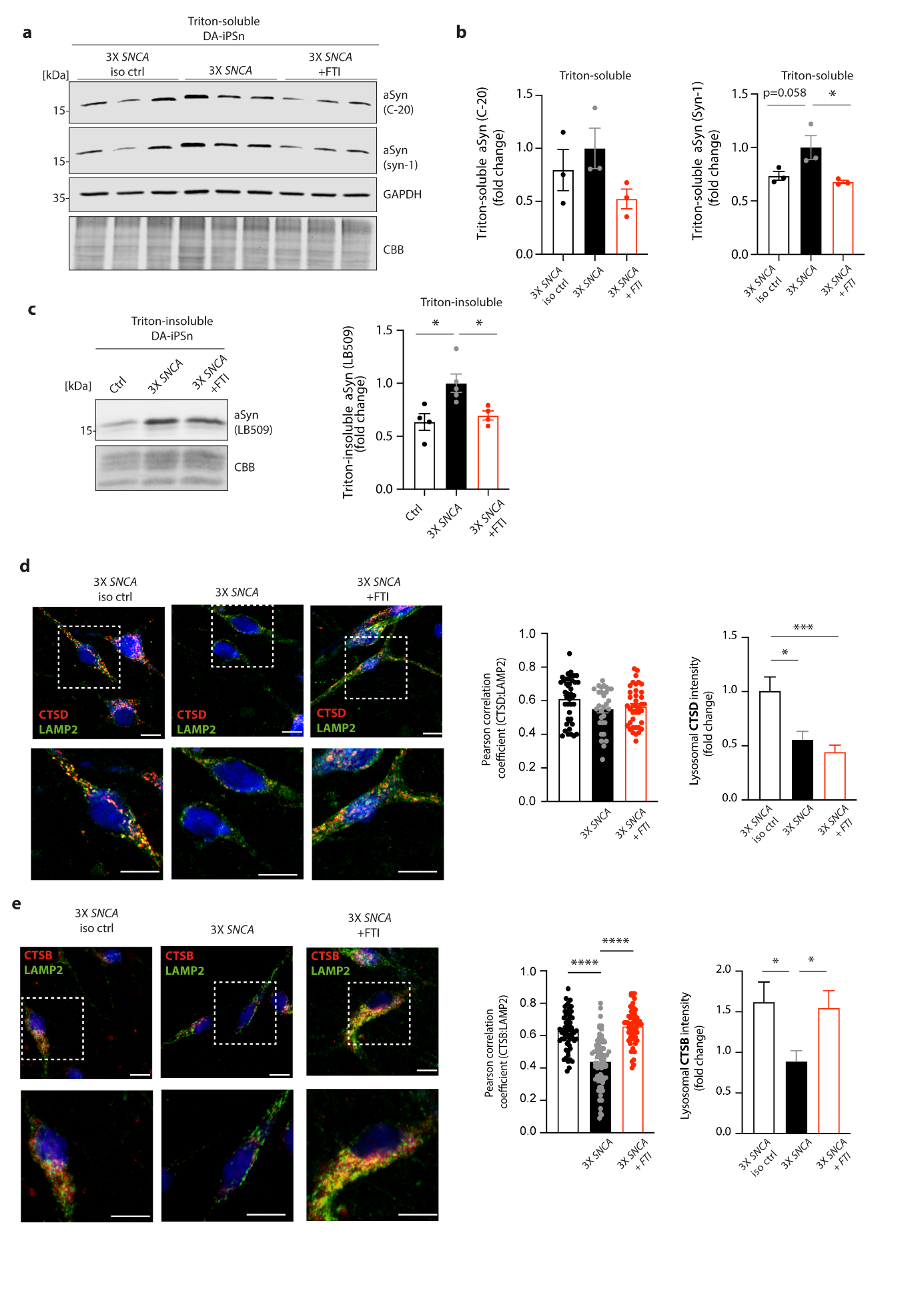
**

**Fig. S6** Improved trafficking of cathepsins by FTI in 3×*SNCA* DA-iPSn.

**a** Western blot analysis of αSyn in Triton-soluble fractions of DA-iPSn by utilizing C-20 and Syn-1 antibodies. 3×*SNCA* neurons were cultured with either DMSO or 5 nM FTI for 7 days with media change every day. 3×*SNCA* iso ctrl treated with DMSO was used as a positive control. GAPDH and CBB served as loading controls.

**b** αSyn quantification of western blot analysis in DA-iPSn 3×*SNCA,* iso ctrl, and 3×*SNCA* treated with 5 nM FTI. Signal intensities of αSyn (obtained with the use of C-20 and Syn-1 antibodies, left and right graph, respectively) were normalized to the GAPDH signal and expressed as fold change, compared to 3×*SNCA* neurons (*n*=3).

**c** Left: Representative western blot analysis of insoluble αSyn in Triton-insoluble fractions of DA-iPSn 3×*SNCA,* iso ctrl, and 3×*SNCA* treated with 5nM FTI. αSyn was detected with the pathology-related LB509 antibody, CBB served as loading control. Right: quantification of western blot analysis of insoluble αSyn in DA-iPSn 3×*SNCA,* iso ctrl, and 3×*SNCA* treated with 5nM FTI. αSyn signals detected with LB509 antibody were normalized to CBB and expressed as fold change, compared to 3×*SNCA* neurons (*n*=4-5).

**d** Left: Representative immunofluorescence images of 3×*SNCA* iso ctrl neurons and 3×*SNCA* DA-iPSn cultured with DMSO or 5 nM FTI. Neurons were stained for CTSD (red) and co-stained with LAMP2 (green). Nucleus is shown in blue. Scale bar: 10 µm. Right: Quantification of CTSD:LAMP2 co-staining by determining Pearson correlation coefficient and lysosomal CTSD intensity analysis in 3×*SNCA* iso ctrl and 3×*SNCA* neurons treated for 7 days with DMSO or FTI (Pearson correlation coefficient and lysosomal CTSD intensity analysis: *n*=15-32 individual cells per group).

**e** Left: Representative immunofluorescence images of 3×*SNCA* iso ctrl neurons and 3×*SNCA* DA-iPSn cultured with DMSO or 5 nM FTI. Neurons were stained for CTSB (red) and co-stained with LAMP2 (green). Nucleus is shown in blue. Scale bar: 10 µm. Right: Quantification of CTSB:LAMP2 co-staining by determining Pearson correlation coefficient, and lysosomal CTSB intensity analysis in 3×*SNCA* iso ctrl and 3×*SNCA* neurons treated for 7 days with DMSO or FTI (Pearson correlation coefficient and lysosomal CTSD intensity analysis: *n*=38-60 individual cells per group).

Statistical analyses were performed by using one-way ANOVA together with Tukey´s posthoc test, with **** *P* < 0.0001, *** *P* < 0.001, * *P* < 0.05.

**
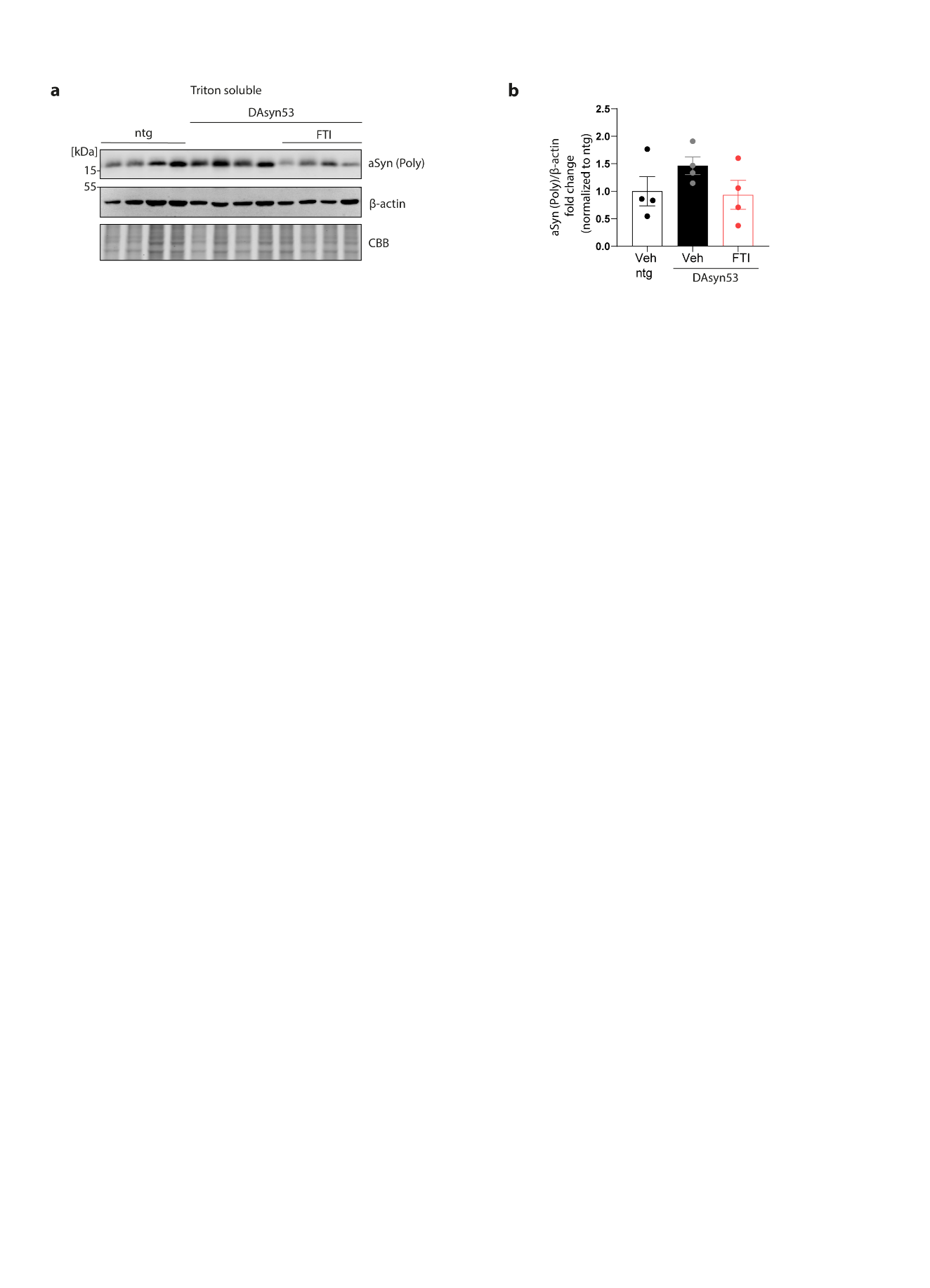
**

**Fig. S7** Farnesyltransferase inhibitor (FTI) treatment decreases the level of soluble αSyn in mice overexpressing αSyn A53T in dopaminergic neurons.

**a** Western blot analysis of soluble αSyn detected with the polyclonal αSyn (Poly) antibody in Triton-soluble fractions of midbrain/thalamic samples of non-transgenic (ntg) mice and mice overexpressing human A53T in dopaminergic neurons (DΑSyn_53_). DΑSyn_53_ mice were treated i.p. with FTI for 26 days. β-actin and CBB served as loading controls.

**b** Quantification of soluble αSyn signal intensities of ntg, DΑSyn_53_ and FTI treated DΑSyn_53_ mice. αSyn signals (detected with polyclonal αSyn antibody (Poly)) were normalized to β-actin signal intensities and displayed as fold change, compared to vehicle ntg mice (*n*=4).

Statistical analysis was performed by using one-way ANOVA together with Tukey’s multiple comparisons test.

**
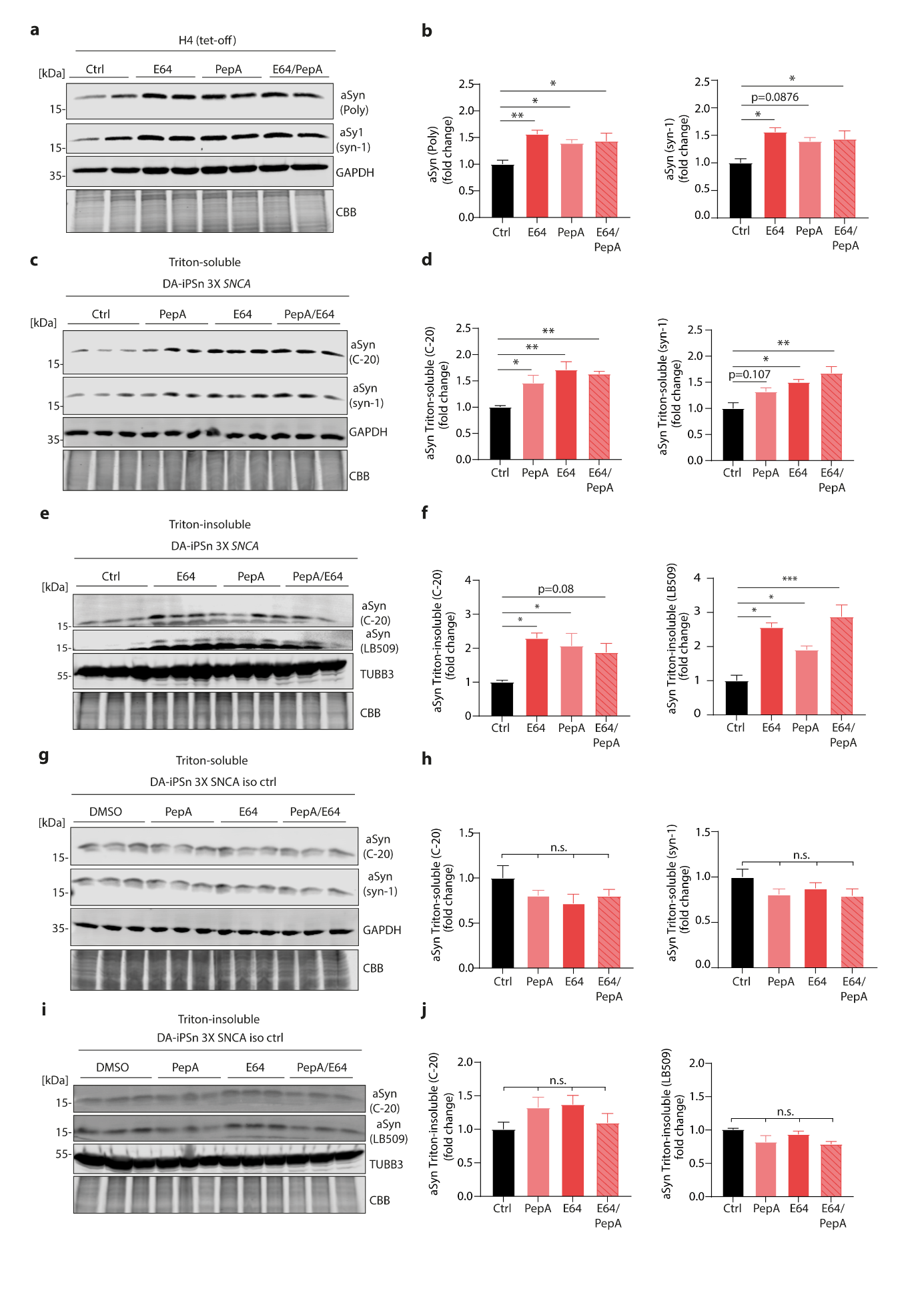
**

**Fig. S8** Inhibition of lysosomal proteases CTSD, CTSL and CTB causes αSyn accumulations.

**a** Representative western blot analyses showing two different αSyn antibodies (Poly and Syn-1) of H4 cells overexpressing αSyn (tet-off) after the inhibition of cysteine cathepsins CTSL and CTSB by E64 and aspartic cathepsin CTSD by Pepstatin A (PepA). Cells were treated for 5 days with every day media change. Control (Ctrl) was treated with the same amount of DMSO. GAPDH and CBB served as loading controls.

**b** Quantification of western blot analyses of αSyn using polyclonal (Poly; left) and Syn-1 (right) antibodies. αSyn signals were normalized to GAPDH and shown as fold change, compared to Ctrl (*n*=3-4).

**c** Western blot analyses of αSyn levels in the Triton-soluble fraction in midbrain dopaminergic (DA) neurons derived from induced pluripotent stem cells (iPSn) harbouring an αSyn triplication (3×*SNCA*). DA-iPSn were treated with CTSD inhibitor PepA and CTSL and B inhibitor (E64) for two weeks with media replacement every second day. Ctrl was treated with the same amount of DMSO. Two different αSyn antibodies were used (C-20 and Syn-1). GAPDH and CBB ensured equal protein loading.

**d** Corresponding analyses of αSyn levels in the Triton-soluble fraction of DA-iPSn using C-20 antibody (left) and Syn-1 antibody (right). Signals were normalized to GAPDH and expressed as fold change, compared to Ctrl (*n*=3).

**e** Western blot analyses of aggregated αSyn in the Triton-insoluble fraction in DA-iPSn 3×*SNCA* with αSyn antibody C-20 and LB509 binding preferably pathological αSyn. Beta-III Tubulin (TUBB3) and CBB served as loading controls.

**f** Corresponding analyses of aggregated αSyn in the Triton-insoluble fraction of DA-iPSn using C-20 antibody (left) and LB509 antibody (right). Signals were normalized to CBB and expressed as fold change, compared to Ctrl (*n*=3).

**g** Western blot analyses of neurons isogenic ctrl of 3×*SNCA* treated with CTSD inhibitor PepA, CTSL and CTSB inhibitor E64 as well as the combination of the inhibitors for two weeks with every second day media replacement. Control cells were treated with DMSO being the dissolving agent of the inhibitors. Immunoblot is showing the Triton-soluble fraction with two different αSyn antibodies (C-20 and Syn-1). GAPDH and CBB served as a loading control.

**h** Corresponding quantification of αSyn signals (C-20, left and Syn-1, right) in the Triton-soluble fraction of DA-iPSn 3×*SNCA* iso ctrl. Signals was normalized to GAPDH and shown as fold change, compared to the Ctrl. (*n*=3).

**i** Western blot analyses of corresponding Triton-insoluble fraction of DA-iPSn 3×*SNCA* iso ctrl. Neurons were treated with PepA, E64 and the combination of both inhibitors. αSyn was detected by C-20 and LB509 antibodies, which predominantly identify pathological αSyn forms. TUBB3 and CBB were used to ensure equal protein load.

**j** Immunoblot quantification with C-20 (left) and LB509 (right). αSyn signals were normalized to CBB and expressed as fold change, compared to Ctrl. (*n*=3).

Statistical analyses were performed by using one-way ANOVA together with Dunnett’s multiple comparison test. *** *P* < 0.001, ** *P* < 0.01, * *P* < 0.05
